# A Mesenchymal Tumor Cell State Confers Increased Dependency on the BCL-X_L_ Anti-apoptotic Protein in Kidney Cancer

**DOI:** 10.1158/1078-0432.CCR-22-0669

**Published:** 2022-11-01

**Authors:** Treg Grubb, Smruthi Maganti, John Michael Krill-Burger, Cameron Fraser, Laura Stransky, Tomas Radivoyevitch, Kristopher A. Sarosiek, Francisca Vazquez, William G. Kaelin, Abhishek A. Chakraborty

**Affiliations:** 1 Department of Cancer Biology, Lerner Research Institute, Cleveland Clinic, Cleveland, OH 44195, USA.; 2 Case Comprehensive Cancer Center, Case Western Reserve University, Cleveland, OH 44106, USA.; 3 Broad Institute of Harvard and MIT, Cambridge, MA 02142, USA.; 4 John B. Little Center for Radiation Sciences, Harvard T.H. Chan School of Public Health, Boston, MA 02115, USA.; 5 Molecular and Integrative Physiology Program, Department of Environmental Health, Harvard T.H. Chan School of Public Health, Boston, MA 02115, USA.; 6 Department of Medical Oncology, Dana-Farber Cancer Institute and Brigham and Women’s Hospital, Harvard Medical School, Boston, MA 02215, USA.; 7 Quantitative Health Sciences, Lerner Research Institute, Cleveland Clinic, Cleveland, OH 44195, USA.; 8 Howard Hughes Medical Institute, Chevy Chase, MD 20815, USA.

## Abstract

**Purpose:**

Advanced/metastatic forms of clear cell Renal Cell Carcinomas (ccRCCs) have limited therapeutic options. Genome-wide genetic screens have identified cellular dependencies in many cancers. Using the Broad Institute/Novartis’ combined shRNA dataset, and cross-validation with the CRISPR/Cas9 DepMap (21Q3) dataset, we sought therapeutically actionable dependencies in kidney lineage cancers.

**Experimental Design:**

We identified preferential genetic dependencies in kidney cancer cells versus other lineages. *BCL2L1*, which encodes the BCL-X_L_ anti-apoptotic protein, scored as the top actionable dependency. We validated this finding using genetic and pharmacological tools in a panel of ccRCC cell lines. Select BCL-X_L_-dependent (versus independent) cell lines were then transcriptionally profiled to identify biomarkers and mechanistic drivers of BCL-X_L_ dependence. Cell-based studies (*in vitro* and *in vivo*) and clinical validations were used to address physiological relevance.

**Results:**

Inactivation of BCL-X_L_, but not BCL-2, led to fitness defects in renal cancer cells, and sensitized them to chemotherapeutics. Transcriptomic profiling identified a ‘BCL-X_L_ dependency’ signature, including an elevated mesenchymal gene signature. A mesenchymal state was both *necessary* and *sufficient* to confer increased BCL-X_L_ dependence. The ‘BCL-X_L_ dependency’ signature was observed in ~30% of human ccRCCs, which were also associated with worse clinical outcomes. Finally, an orally bioavailable BCL-X_L_ inhibitor, A-1331852, showed anti-tumor efficacy *in vivo*.

**Conclusions:**

Our studies uncovered an unexpected link between cell state and BCL-X_L_ dependence in ccRCC. Therapeutic agents that specifically target BCL-X_L_ are available. Our work justifies testing the utility of BCL-X_L_ blockade to target, likely, a clinically aggressive subset of human kidney cancers.

## INTRODUCTION

Renal Cell Carcinoma (RCC), among the top ten forms of cancer in humans, is expected to cause ~14,000 deaths this year in the USA (1). Although early stage RCC, which is observed in nearly 60% of patients, can be effectively managed with either surgery or surveillance, advanced or metastatic RCC is associated with poorer clinical outcomes. Loss of the von Hippel-Lindau tumor suppressor protein (pVHL) is the hallmark of clear cell Renal Cell Carcinomas (ccRCCs), which represent ~75% of RCCs (2). Therefore, mechanistic studies defining the consequences of pVHL loss have informed the pre-clinical discovery of actionable dependencies in ccRCC.

pVHL functions in an E3 ligase complex that is, perhaps, best known to mediate oxygen-dependent destruction of the α-subunit of the Hypoxia Inducible Factor (HIFα) (3, 4). Consequently, HIFα accumulates in pVHL-deficient ccRCCs. Among the two transactivation-competent isoforms of HIFα, HIF1α and HIF2α, the chronic activation of HIF2α, in particular, drives ccRCC tumorigenesis (5, 6). Therapeutic strategies that inactivate HIF2α have been developed in the last decade (7, 8). These approaches have shown promise in both pre-clinical (9, 10) and clinical settings (11). Besides HIF2α, targeting Receptor Tyrosine Kinases in combination with checkpoint blockade, has recently emerged as a viable option for disease management (12). But not all patients respond, and not all responses are durable. Therefore, new treatments, especially to target tumors that are refractory to current therapeutics, are urgently needed.

Recent largescale screening efforts, such as the Broad Institute’s shRNA Achilles (13) and CRISPR/Cas9 DepMap screening projects (14), and Novartis’ DRIVE project (15), have enabled the identification of novel oncogenic dependencies. CRISPR/Cas9-based screens routinely yield phenotypes associated with near complete elimination (knockout) of the gene product. In contrast, shRNA screens often score phenotypes associated with partial mRNA reduction (knockdown), and perhaps better model the incomplete blockade of target proteins that is typically achieved with drugs. Importantly, though, the candidates identified through shRNA-based approaches need to be rigorously vetted because of the notorious “off-target” cytotoxicity associated with shRNA use (16).

Here, we reasoned that the genetic lesions acquired during renal oncogenesis and/or the master regulators of lineage specification could be targetable dependencies in ccRCC. Therefore, using genetic dependency databases, we sought to identify kidney lineage-specific dependencies (versus other cells), hoping some of these could be actionable targets in kidney cancer.

## MATERIALS AND METHODS

### Cell Lines

A-498, CAKI-2, 769-P (RRID:CVCL_1050), and 786-O (RRID:CVCL_1051) cells were obtained from American Type Culture Collection. RCC4 (RRID:CVCL_0498) cells were a kind gift from Dr. Peter Ratcliffe’s laboratory (Oxford University). UMRC-2, UMRC-6, and UOK101 (RRID:CVCL_B076) cells were obtained from Dr. Bert Zbar and Dr. Martson Linehan (National Cancer Institute). OSRC2 (RCB0735, RRID:CVCL_1626), TUHR4TKB, and TUHR10TKB (RCB1275, RRID:CVCL_5952) cells were obtained from the Riken cell culture collection. The SU-DHL-6 (KCB 2016029YJ-A, RRID:CVCL_2206) cells were obtained from Dr. Alexandru Almasan and Dr. Neetu Gupta (Cleveland Clinic). Cells were maintained in the following media: A-498, RCC4, UMRC-2, UMRC-6, UOK101, and 786-O in Dulbecco’s Modified Eagles Medium (DMEM) (Life Technologies 11995073); OSRC2, SU-DHL-6, TUHR4TKB, and 769-P cells in RPMI-1640 (Life Technologies 11875119); and, CAKI-2 cells in McCoy’s 5A medium (Life Technologies 16600108). All media was supplemented with 10% FBS and 1X Penicillin-Streptomycin. Lentivirally transduced cells were selected with Puromycin (2 µg/ml) or Blasticidin (10 µg/ml), as appropriate. All cells were grown at 37°C in 5% CO_2_.

### Identification of Kidney-selective Dependencies

To find kidney dependencies, we filtered the DepMap 21Q3 sample information file for kidney cell lines (https://depmap.org/portal/download/). Rhabdoid (G401, JMURTK2) and transitional cell lines (BFTC909) were removed, resulting in 32 total kidney cell lines, 26 of which were included in the DEMETER2-Combined RNAi dependency dataset (17). We sought strong gene dependencies (DEMETER2 < −1, the median of positive controls), that scored in at least three kidney cell lines. We removed ‘pan-dependent’ genes that were scored dependencies in over 90% cancer cell lines (18). The remaining 194 strong kidney dependencies were ordered by LRT score (15, 19) (likelihood ratio test of skewed-t vs symmetric gaussian fit of each gene dependency distribution across all pan-cancer cell lines), which meant that strong dependencies on higher-ranking genes were less likely to be from the tail of a null distribution. Finally, *BCL2L1* gene effects from the DEMETER2-Combined RNAi dataset were compared to the DepMap 21Q3 (Cancer Dependency Map Portal, RRID:SCR_017655) CRISPR/Cas9 Achilles dataset for independent validation.

### Plasmids

The shRNA constructs (Sigma) were as follows: BCL-2 (TRCN0000040069, TRCN0000010303, and TRCN00000293675); BCL-X_L_ (TRCN0000033499, TRCN0000033500, and TRCN0000033503); and Control (SH001 and SH002). BCL-X_L_, amplified from a GFP-BCL-X_L_ fusion (20), was sub-cloned into pDONR223 (RRID:Addgene_60532) using Gateway cloning (Invitrogen). Rescue constructs (499R and 499/500R) were generated by sequentially introducing silent mutations using site-directed mutagenesis (Quickchange II XL; Agilent 200521), first at the sh499 site and then, using this construct as a template, the sh500 site. Expression vectors were generated by Gateway cloning into the lentiviral pLX304 destination vector (RRID:Addgene_25890). The pLenti-CMV-Puro constructs to express TWIST, was described previously (21).

### Lentiviral Transduction

Lentiviruses were packaged by transfecting 293FT cells (ThermoFisher, R70007) with Lipofectamine 2000 (Invitrogen 11668019), seeded overnight with 1.8 × 10^6^ cells in 6 cm dishes. Transfection mixes had 1.5 µg lentiviral plasmid, 1.5 µg of helper plasmids [psPAX2 (RRID:Addgene_12260):pMD2.G (RRID:Addgene_12259); 3:1 ratio], and 9 µl Lipofectamine 2000, in OptiMEM. Sixteen hours later, media was changed, and viral supernatant was collected at 48 and 72 hours post transfection and combined. The lentivirus-containing supernatant was filtered through a 0.45 μm filter and frozen at −80°C. For infections, 250 μl of lentivirus was added to cells (75,000/well, seeded overnight into 12-well plates, 8 μg/ml polybrene), and the plates were centrifuged at 300× *g* for 40 min. Lentivirus was removed after eight hours and the cells were grown for 24 hours before selection.

### Flow Cytometry

All flow cytometry was analyzed with FlowJo v10.4.2 (RRID:SCR_008520). For Annexin V versus PI staining, cells (150,000 cells/well, seeded overnight, 6-well plates) were treated with A-1331852 for 16 hours (100 nM for A-498, UMRC-2, and UMRC-6; 10 nM for CAKI2). Floating and adherent cells were harvested and stained using the APC Annexin V Apoptosis Detection Kit with PI (640932, BioLegend), as per the manufacturer’s instructions.

For CD44 measurements, ccRCC cells [typically grown for 3 days and harvested at <90% confluency using the TrypLE reagent (Gibco)], were washed in 1% BSA/PBS, and stained using an APC-conjugated CD44 antibody (Cell Signaling Technology Cat# 80813, RRID:AB_2799964). Typically, 1.5 ×10^6^ cells with 1:100 anti-CD44 in 100 µl volume of 1% BSA/PBS, at RT/45 mins.

### Immunoblot Analysis

Cell were lysed in lysis buffer [50 mM Tris.Cl (pH 7.5), 400 mM NaCl, 1% Nonidet P-40, 1 mM EDTA, and 10% glycerol], freshly supplemented with a protease inhibitor cocktail (cOmplete Mini, Roche). Proteins were resolved by SDS-PAGE using 8%, 10%, or 12% polyacrylamide gels, as relevant, and transferred onto 0.2 µm nitrocellulose membranes. As a loading control, for blots with multiple ccRCC cell lines, membranes were stained with Ponceau S solution (Sigma P3504), RT/ 5 mins, and imaged, before immunoblotting.

The following antibodies were used: BCL-X_L_ (Cell Signaling 2764, 1:1000), BCL-2 (Cell Signaling Technology Cat# 4223, RRID:AB_1903909, 1:1000), p53 : (Abcam Cat# 5000-1, RRID:AB_514418, 1:1000), phospho-p53 (Ser15) (Cell Signaling Technology Cat# 9284, RRID:AB_331464, 1:1000), p21 (Santa Cruz Biotechnology Cat# sc-397, RRID:AB_632126, 1:1000), Vinculin (Cell Signaling Technology Cat# 13901, RRID:AB_2728768, 1:1000), V5 Tag (Cell Signaling 80076, 1:1000), and HRP-conjugated secondary antibodies (Pierce, 1:5000). Chemiluminescent HRP substrates (Supersignal West Pico PLUS; Thermo Fisher Scientific; Pierce ECL Plus, Thermo Fisher Scientific; or Immobilon ECL Ultra, Millipore) were used.

### Cell Viability Assays

ccRCC lines were seeded overnight (CAKI-2 and TUHR4TKB: 4,000 cells/well; Others: 2,000 cells/well; in 96-well plates) and then treated with the respective inhibitors. Cells were exposed for 72 hours to a six-point, three-fold serial dilution of ABT-263: 1.1 to 10 μM; A-1331852: 0.02 to 2 μM; and ABT-199: 0.02 to 2 μM. Cell viability was analyzed using the XTT assay (Cell Proliferation Kit II, Roche) or CellTiter-Glo (Promega), per manufacturer’s instructions. IC_50_ values were determined though non-linear regression (curve fitting) using Graphpad Prism (RRID:SCR_000306). For crystal violet staining, cells were washed with 1X PBS and stained with 0.4% crystal violet in 20% methanol for 30 minutes, washed twice with 1X PBS, air-dried overnight, and scanned.

### Drug-drug Interaction Assay and Analysis

UMRC-2 and OSRC2 cells were seeded at 2,000 cells/well in 96-well plates and treated with compound combinations. Docetaxel and Doxorubicin: five-point, three-fold serial dilution, 2 to ~222 nM; 5-Flurororuacil: five-point, three-fold serial dilution, 0.07 to 2 μM; A-1331852: two concentrations, 670 nM and 2 μM. After 72 hours, cell viability (relative to DMSO control) was measured by XTT. Synergy was calculated using the SynergyFinder (RRID:SCR_019318) web application (22) with default parameters.

### RNA-Seq and Gene Set Enrichment Analysis (GSEA)

Total RNA was extracted using Trizol (Life Technologies), quantified using the Qubit RNA Assay Kit (Life Tech), and quality was determined on the Bioanalyzer using the RNA Pico Kit (Agilent). cDNA libraries were prepared using the Collibri Stranded RNA Library Prep Kit for Illumina with 50–100 ng of total RNA, following the manufacturer’s protocol. Prepared DNA libraries were quantified using the Qubit High Sensitivity DNA Kit (Life Tech) and library sizes determined using the Bioanalyzer High Sensitivity Chip Kit (Agilent). Finally, libraries were subjected to qPCR using the Universal Library Quantification Kit for Illumina (Kapa Biosystems) and a 7900HT Fast qPCR (ABI) instrument. Libraries passing all QC criteria were sequenced on the NextSeq 550 Sequencing System (Illumina) at a final concentration of 12 pM on a single read flowcell with 75 sequencing cycles.

The base-called sequences were obtained using the DRAGEN Bio-IT Platform. Raw sequences (.fastq files) were QC tested using FastQC and had, on average, high-quality metrics (>30 Phred score) and nucleotide distributions. Reads with Phred scores <28 were trimmed using Cutadapt (v1.16). Reads were aligned to the UCSC hg38 build of the human transcriptome using HISAT2 (v2.1.0) and total read counts per gene were measured using featureCounts (v1.6.4). Counts were filtered, normalized (Trimmed Mean of M-values), and differential expression was determined using EdgeR (v3.12).

GSEA was performed on the EdgeR normalized counts, using software downloaded from http://www.broad.mit.edu/gsea/downloads.jsp. Gene Sets with an NES>1.5; FDR<0.10; and a nominal *p*<0.05 were considered significant.

### Patient Tumor Samples from TCGA

HTSeq counts files for clear cell (TCGA-KIRC) renal cell carcinomas were downloaded from the Genomic Data Commons Data Portal. Counts were filtered and normalized using EdgeR (RRID:SCR_012802). Principal component analysis was performed on ccRCC tumors using the differentially expressed genes identified between sensitive (A-498 and CAKI-2) and insensitive (OSRC2 and UMRC-2) RCC cell lines. K-means and t-distributed stochastic neighbor embedding methods were performed using the top principal components, which explained 75% of the variation. Clusters were determined by selecting the optimal within-clusters sum of squares (elbow method) and annotated by their similarity (reciprocal of Euclidean distance) to sensitive or insensitive cell line derived GSEA profiles.

### Response to A-1331852 by Cell State Perturbations

Cells were pretreated for 3 days with ATRA (1 µM) or TGFβ (10 ng/ml), as relevant. After confirmation of cell state changes (using CD44), cells were seeded into 96-well plates in media containing ATRA or TGFβ, as appropriate. The next day, media was aspirated, and cells were cultured for 7 days with A-1331852 (~14 nM to 10 µM). Viability was evaluated using CellTiter-Glo (Promega). All changes were normalized to the untreated (DMSO) control in each experimental arm to normalize for any inadvertent differences in cell doubling.

### In Vivo Studies

Sub-cutaneous tumors were measured weekly using calipers and volumes were calculated as (length x width^2^)/2. A-1331852 was synthesized in bulk and purchased from Chem-Space. Oral formulations were prepared, as previously described (23), and animals were dosed twice a day, 25mg/kg, for up to 4 weeks. Tumor and normal tissues were harvested upon necropsy for histological analysis. All animal experiments were approved by Cleveland Clinic’s IACUC (protocol no. 0002168).

## DATA AND MATERIALS AVAILABILITY

All the data necessary to evaluate the conclusions of the manuscript are provided in the paper and/or the Supplementary Materials. Gene expression data is deposited into the Gene Expression Omnibus [(GEO), RRID:SCR_005012, GSE173618]. All reagents are available commercially and published constructs will be deposited in Addgene to ensure public distribution.

## RESULTS

### BCL-X_L_ is a Lineage-specific Dependency in Kidney Cancer

To identify dependencies that are selectively enriched in kidney cancer cells (versus all other lineages), we used the Broad Institute/Novartis’ (shRNA) combined dataset. This dataset reports changes in shRNA abundance over 6–8 weeks following lentiviral transduction of a pooled genome-wide shRNA library into a large collection of cancer cells, representing various cancer types and lineages (13). To avoid losing critical dependencies due to inherent genetic heterogeneity between cells (i.e. false negatives), we sorted dependencies that showed strong selectivity [Likelihood Ratio Test (LRT)>100] in at least a subset of kidney lineage cells. We excluded “common essential genes”, which were known dependencies in >90% of all cancer cells (18). Finally, we confirmed that the scored dependency was observed in a minimum of three independent kidney cell lines. These strategies scored ~twenty potential dependencies (Supplementary Table S1).

**FIGURE 1.**
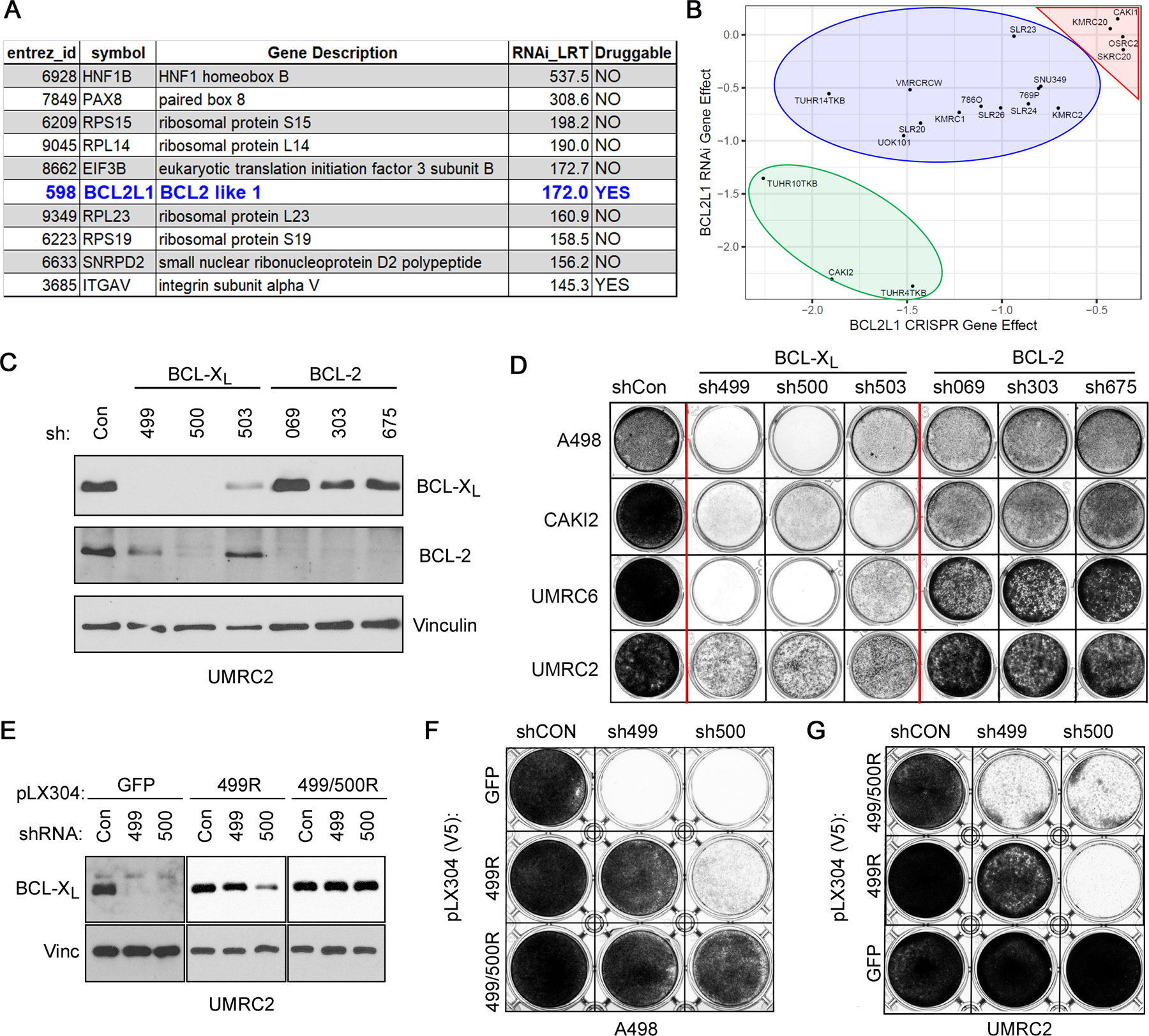
BCL-X_L_ is a Strong Dependency in a Subset of Kidney-lineage Cancer Cells. **A,** List of dependencies enriched for selectivity in a subset of kidney lineage cells, as indicated by Likelihood Ratio Test (LRT) scores > 100. **B,** Comparison of BCL-X_L_ dependency in the RNAi versus CRISPR/Cas9 dependency maps, in the indicated kidney cancer cell lines. Negative scores indicate a dependency with scores < −1 (strong, green oval), −0.5 to −1.0 (intermediate, blue oval), and >−0.5 (neutral/resistant, red triangle), annotated based on the CRISPR/Cas9 dataset. Immunoblots (**C**) and crystal violet staining (**D**) of the indicated cell lines that were lentivirally transduced to express shRNAs targeting the indicated BCL-2-family genes or non-targeting controls (Con). Immunoblots (**E**) and crystal violet staining (**F** and **G**) of the indicated cell lines that were lentivirally transduced to express the indicated shRNA-resistant versions of BCL-X_L_ or GFP, as a control, followed by lentiviral expression of the indicated shRNAs. (**C**) and (**E**) were done 3 days post infection with the shRNA-expressing lentiviral particles. (**D**), (**F**), and (**G**) were done 3 days post selection for A-498, CAKI-2, and UMRC-6 cells, and 7–10 days post selection for UMRC-2 cells.

*HNF1B* and *PAX8*, both of which have an established role in kidney lineage specification (24, 25), scored among our top ten “hits”, ranked by LRT (Fig. 1A). Many of the scored candidates were not druggable (e.g. ribosomal subunits); however, *BCL2L1*, scored as the top gene that encoded a druggable product. Comparing RNAi data with the CRISPR/Cas9 dependency maps (14), we confirmed that several ccRCCs (e.g. CAKI-2, TUHR4TKB, and TUHR10TKB) showed strong *BCL2L1* dependence in both datasets (Fig. 1B), independently validating our initial observations.

*BCL2L1* encodes the anti-apoptotic BCL-X_L_ protein (26), a member of the BCL-2 protein family (27). To confirm our initial observations, we lentivirally transduced representative human ccRCC cell lines (A-498, CAKI-2, UMRC-2, and UMRC-6) with three shRNAs that targeted either *BCL2L1* (499, 500, and 503), *BCL2* (069, 303, and 675), or a non-targeting control (shCon). Unlike *BCL2* loss, which was well tolerated, *BCL2L1* knockdown caused evident cytotoxicity in A-498, CAKI-2, and UMRC-6 within 7 days and in UMRC-2 cells ~15 days post infection (Fig. 1C and D).

Two BCL-X_L_ targeting shRNAs (499 and 500) also partially downregulated BCL-2. To formally establish that the cytotoxic effects of these *BCL2L1* shRNAs were “on-target”, we engineered a BCL-X_L_ cDNA that harbored silent mutations in the sh499 recognition sequence. Expression of this construct (499R) rescued the cytotoxicity associated with sh499, but not sh500 (Fig. 1, E – G). Introducing additional mutations into the 499R backbone to also make it resistant to sh500 (499/500R) rescued the cytotoxicity associated with both sh499 and sh500 (Fig. 1, E – G). Together, these studies confirmed that a subset of ccRCCs are highly dependent on BCL-X_L_, but less so on BCL-2. Moreover, certain ccRCCs (e.g. UMRC-2) presented with delayed cytotoxicity in response to BCL-X_L_ loss.

### Pharmacological BCL-X_L_ Blockade Mimics Results of Genetic Studies

Pharmacological strategies to target the BCL-2 family proteins have identified the BCL-X_L_/BCL-2 dual inhibitors, ABT-737 (28) and ABT-263 (Navitoclax) (29); the BCL-2-specific inhibitor, ABT-199 (Venetoclax) (30); and more recently the BCL-X_L_-specific inhibitor, A-1331852 (31). A-1331852 exhibits a 600-fold specificity for BCL-X_L_ (versus BCL-2) and readily kills the BCL-X_L_-dependent MOLT-4 cells (EC_50_: 6.3 nM), but not the BCL-2-dependent RS4 cells (EC_50_: >5000 nM) (31).

**FIGURE 2.**
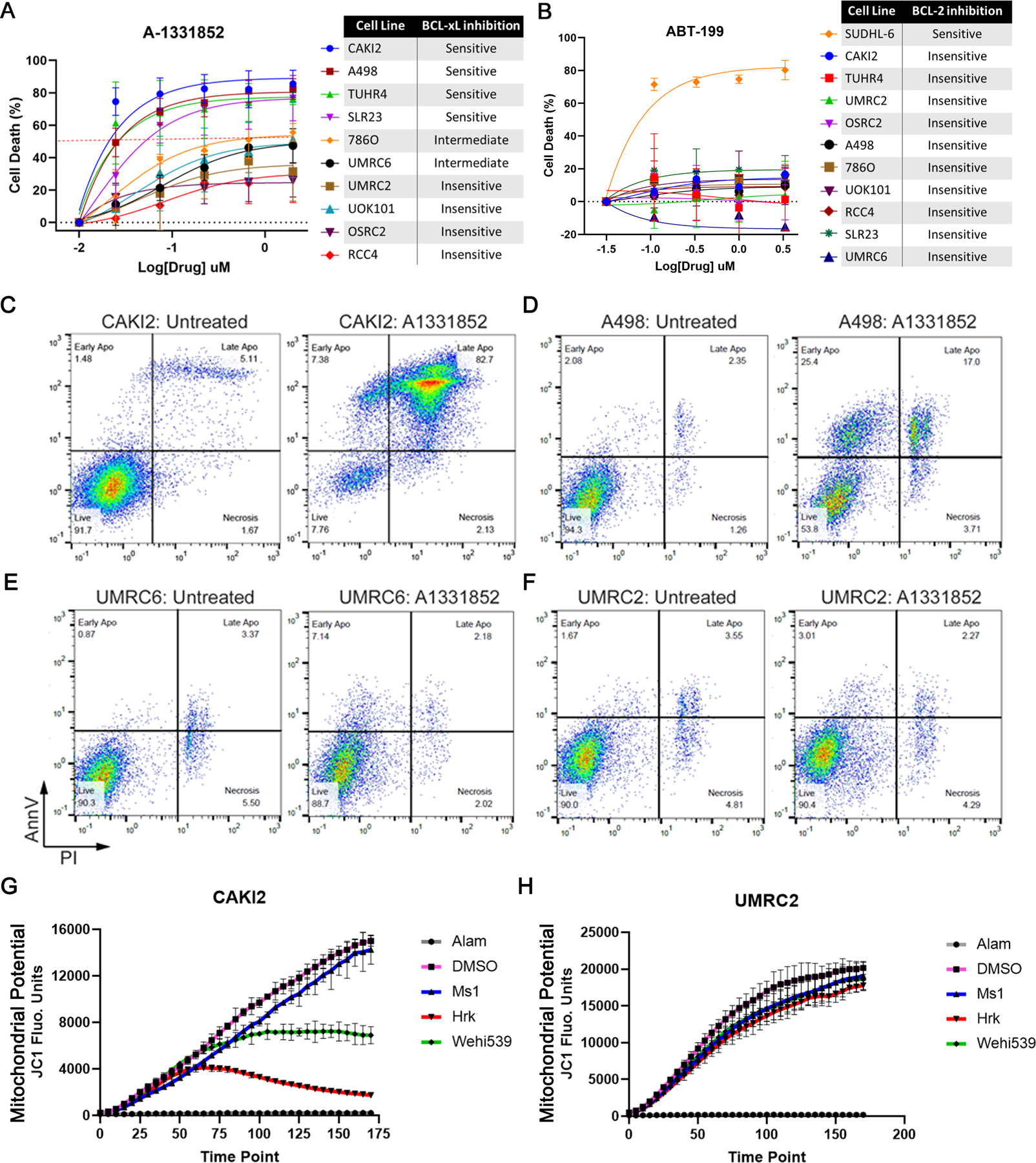
BCL-X_L_ Inhibition Promotes Apoptotic Cell Death in a Subset of ccRCCs. Percent cell death, relative to the DMSO-treated control cells, determined using the XTT assay in the indicated ccRCC cell lines that were treated with the indicated concentrations of the BCL-X_L_ inhibitor A-1331852 (**A**) or the BCL-2 inhibitor ABT-199 (**B**) for 3 days. Flow cytometric analysis to compare AnnexinV-FITC (AnnV) versus Propidium Iodide (PI) staining in CAKI-2 (**C**), A-498 (**D**), UMRC-6 (**E**), and UMRC-2 (**F**) cells that were treated with A-1331852 or DMSO (Untreated control), as indicated. In (**C**) cells were treated with 10 nM A-1331852 for 16 hours; whereas, in (**D**), (**E**), and (**F**) cells were treated with 100 nM A-1331852 for 36 hours. JC1 fluorescence measurement at the indicated time-points in CAKI-2 (**G**) and UMRC-2 cells (**H**) that were exposed to Alamethicin (Alam) (positive control), DMSO (negative control), sensitizer BH3 peptides [MS1 (30 µM) targeting MCL-1 and Hrk (100 µM) targeting BCL-X_L_], or the small molecule BCL-X_L_ inhibitor Wehi539 (1 µM).

To validate our genetic studies, we treated a panel of human ccRCC lines with BCL-2 family inhibitors. We observed that cell lines that were exquisitely sensitive to BCL-X_L_ loss in genetic studies (e.g. CAKI-2 and TUHR4TKB) were also highly sensitive to acute treatment with either ABT-263 (Supplementary Figs. S1A and S1B) or the BCL-X_L_-specific inhibitor A-1331852, with cellular IC_50_ values in the low nM range (Fig. 2A; Supplementary Fig. S1C). In contrast, cell lines like UMRC-2, which showed delayed response to genetic BCL-X_L_ loss, were likewise less responsive to three days of BCL-X_L_ blockade (Fig. 2A; Supplementary Fig. S1, A – C). These analyses also identified the A-498 and SLR23 cells as additional examples of A-1331852 sensitive ccRCCs.

In contrast to the BCL-2-dependent B-cell lymphoma line SU-DHL-6, the ccRCC lines were virtually resistant to the BCL-2 inhibitor ABT-199 (Fig. 2B; Supplementary Fig. S1D). Moreover, as expected from A-1331852’s BCL-X_L_ specificity (31), SU-DHL-6 cells showed virtually no response to A-1331852 at concentrations that were potently cytotoxic for the ccRCCs (Supplementary Fig. S1E).

Relying on structural models (Supplementary Fig. S1F), we noted three critical residues - F97, Y101, and Y195 – in the P2 pocket in BCL-X_L_. Mutations in all three residues (e.g. F97W, Y101H, and Y195F) retained functionality, as evidenced by their ability to rescue the cytotoxicity associated with the BCL-X_L_ 499 shRNA (Supplementary Figs. S1G and S1H); however, F97W (and to a lesser extent, Y195F) protected against the cytotoxic effects of A-1331852 (Supplementary Figs. S1I and S1J). Altogether, we concluded that the effects of A-1331852 were largely driven by BCL-X_L_ blockade and that nearly 40% of the tested ccRCC lines were sensitive (IC_50_ in the nM range) to A-1331852. Perhaps, because of underlying genetic differences, sensitivity in only a subset of cell lines is a common occurrence in pre-clinical studies. For example, the HIF2α inhibitor, which is now FDA approved for use against ccRCC, efficiently targeted only ~30% cell lines in pre-clinical studies (9, 10).

We next confirmed that the cytotoxicity triggered by A-1331852 treatment occurred because of increased apoptosis. Upon A-1331852 treatment, using Annexin V (AnnV) versus Propidium Iodide staining, we found a significant increase in the apoptotic population in the BCL-X_L_-inhibitor sensitive CAKI-2 and A-498 cells, but not the relatively insensitive UMRC-2 and UMRC-6 cells (Fig. 2, C – F).

Anti-apoptotic BCL-2 family proteins function by binding and sequestering pro-apoptotic “BH3-only” proteins (e.g. BIM, BID, and PUMA) or the pore-forming proteins (e.g. BAX and BAK). Synthetic, pro-apoptotic BH3 peptides can mimic or block these interactions and trigger cellular apoptotic programs, which can be probed using hallmarks of apoptosis (e.g. mitochondrial membrane depolarization) (32). Using “BH3 profiling”, we compared ccRCC’s dependency on the BCL-2 family proteins. In line with our pharmacological experiments, we observed that the BCL-X_L_-dependent CAKI-2 cells showed significant mitochondrial depolarization (indicated by loss of JC1 fluorescence) with BCL-X_L_ blockers, such as the Hrk peptide and the BCL-X_L_ inhibitor, Wehi539, but not with MCL-1 blockers (e.g. MS1 peptide) (Fig. 2G). In contrast, the UMRC-2 cells, which were less responsive to acute BCL-X_L_ inhibition, were non-responsive to BCL-X_L_ blockers (Fig. 2H). These results demonstrated that BCL-X_L_ actively restrains pro-apoptotic signaling in ccRCCs.

### BCL-X_L_ Inhibition Sensitizes Kidney Cells to Chemotherapeutic Agents

RCCs are known for their resistance to traditional chemotherapeutics (33), which typically function by promoting apoptosis (32). We hypothesized that BCL-X_L_ function, which drives therapeutic resistance in many cancers, is a physiological barrier to chemotherapeutic response in ccRCC. To address this hypothesis, we treated the A-1331852-insensitive UMRC-2 and OSRC2 cells with various combinations of A-1331852 and chemotherapeutics such as 5-Fluorouracil (5-FU), Docetaxel, and Doxorubicin. To measure potential synergy or antagonism, we modeled cell viability using SynergyFinder Plus (22), a visualization package that integrates four drug-drug interaction models (e.g. HSA, Loews, Bliss, and ZIP) (34). This analysis demonstrated synergy at many combinations of the BCL-X_L_ inhibitor and all three chemotherapy drugs, and using all the models (Supplementary Fig. S2; Supplementary Table S2). Therefore, BCL-X_L_ inhibition sensitized ccRCCs to several chemotherapeutic agents.

### Epithelial-Mesenchymal Transition (EMT) is Associated with BCL-X_L_ Dependency

We began our search for potential biomarkers of BCL-X_L_ dependence in cells by comparing the expression of BCL-X_L_ and BCL-2. Unfortunately, these studies showed that (**a**) mRNA levels of *BCL2L1* correlated poorly with BCL-X_L_ DEMETER dependency scores (r^2^ < 0.3, Pearson’s correlation coefficient; Supplementary Fig. S3A); (**b**) neither the protein expression of BCL-X_L_ or BCL-2 (Supplementary Fig. S3B), nor the relative abundance of BCL-2 and BCL-X_L_ (Supplementary Fig. S3, C – E), were sufficient to predict BCL-X_L_ dependency; and, (**c**) there were no discernible differences in the expression of different BCL-X isoforms in ccRCC, with BCL-X_L_ being the predominant species in both sensitive and insensitive ccRCC lines (Supplementary Fig. S3F).

To identify determinants of BCL-X_L_ dependency in an unbiased manner, we performed transcriptomics studies. We chose two representative cells that were sensitive (S: A-498 and CAKI-2) and two that were insensitive (I: OSRC-2 and UMRC-2) to acute BCL-X_L_ blockade. We then transcriptionally profiled, using RNA-Seq, these cells under (untreated) native conditions and after acute treatment with A-1331852, under experimental conditions that preceded any overt cytotoxicity, which we feared could confound our gene expression studies.

**FIGURE 3.**
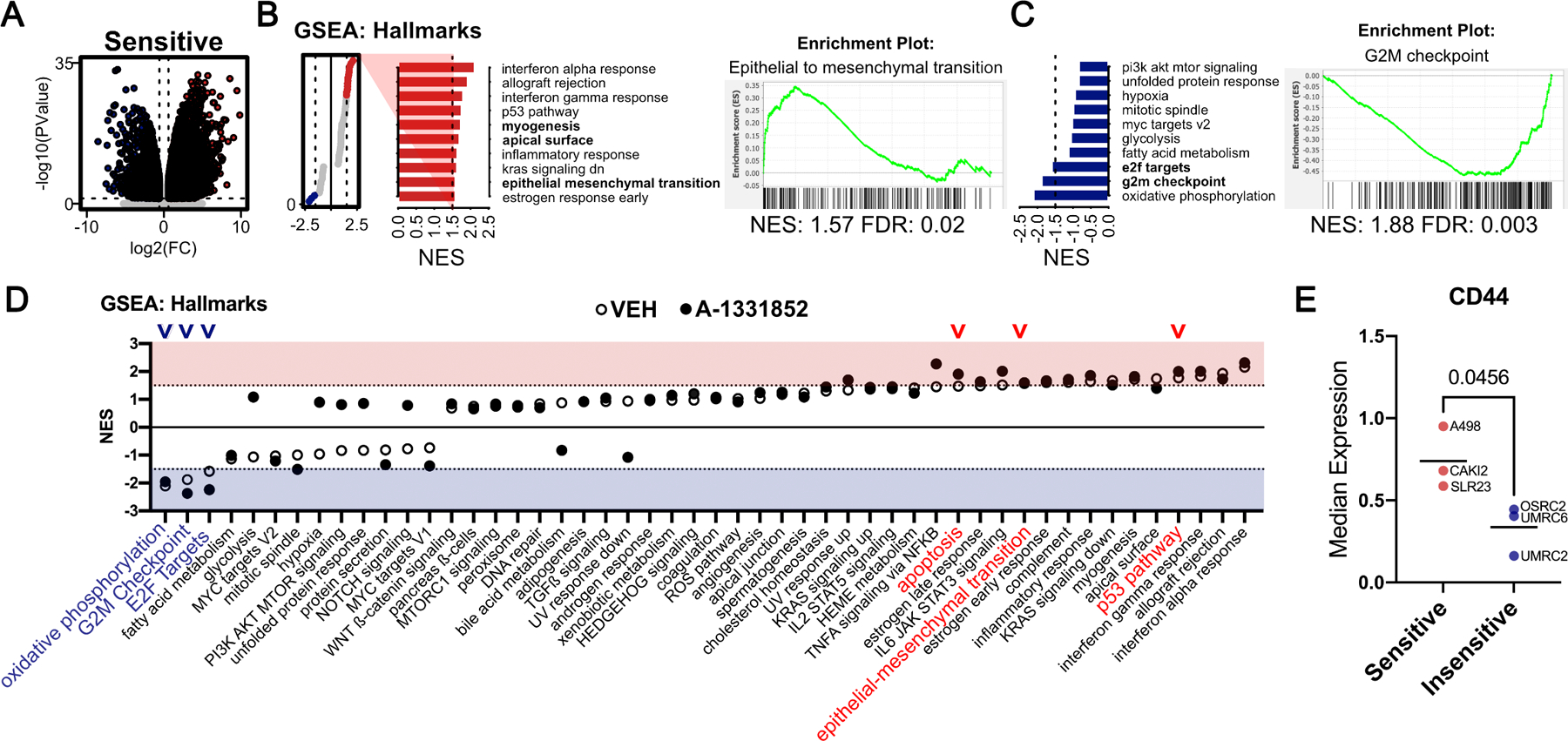
Transcriptomics Analysis Identifies Correlates Associated with BCL-X_L_ Dependency. **A,** Volcano plot of genes that were identified as differentially expressed (bottom, ≥ 1.5-fold and FDR ≤ 10%, as determined by RNA-Seq) in BCL-X_L_ inhibitor ‘Sensitive’ (S) versus ‘Insensitive’ (I), under untreated basal conditions. Gene-sets that were enriched in Sensitive (**B**) or enriched in Insensitive (**C**) ccRCC cells (NES ≥ 1.5 and FDR ≤ 10%), under untreated conditions with representative plots of the indicated gene sets. **D,** Lollipop plot depicting results of GSEA (NES ≥ 1.5 and FDR ≤ 10%) comparing RNA-Seq data from S vs I cells that were either untreated (open circles) or A-1331852 treated (filled circles) ccRCC cells. Arrowheads mark key differential gene sets. **E,** CD44 levels, as determined by flow cytometry, in the indicated BCL-X_L_ inhibitor Sensitive and Insensitive cell lines.

We analyzed the transcriptional signatures using EdgeR and first identified differentially expressed genes (DEGs) in the sensitive (S) versus insensitive (I) lines under untreated conditions (Fig. 3A; Supplementary Table S3). We noted that the expression of certain BCL-2 related genes, including *BCL2L11* (which encodes BimL), *BIK*, and *BAK1*, were elevated in the BCL-X_L_-dependent lines (Supplementary Fig. S3G). Moreover, analysis of the DEGs against annotated gene sets described in mSigDB (35), using Gene Set Enrichment Analysis (36), showed prominent differences in untreated sensitive cells versus their A-1331852-insensitive counterparts (Fig. 3B and C; Supplementary Table. S4), including differences in apoptosis, p53 response, cytokine response (e.g. interferon response), and cell state [e.g. epithelial to mesenchymal transition (EMT)] pathways (Fig. 3, B – D; Supplementary Table S5).

A-1331852-sensitive cells mounted a stronger transcriptional response to acute BCL-X_L_ blockade, as compared to the insensitive cells (Supplementary Fig. S3H). This transcriptional response further amplified the pathway-level differences that already existed between the sensitive versus insensitive cells, including the apoptotic pathway (Fig. 3D; Supplementary Table S5). We found that nine overlapping gene sets, which predominantly included cytokine response pathways, were induced in response to A-1331852 in both sensitive lines (Supplementary Fig. S3I; Supplementary Table S6). A-1331852 treatment also triggered transcriptional induction of many shared genes between the two sensitive lines (Supplementary Fig. S3J; Supplementary Table S7), including EGR family genes and the Notch-target HEY1. The combinatorial effects of these changes likely drive A-1331852 response.

To identify predictors of BCL-X_L_ dependence, we focused on the biological pathways that were inherently different in untreated sensitive versus insensitive lines. Activation of p53 triggers cell death via multiple pro-apoptotic proteins (37, 38), including certain BCL-X_L_-dependent pathways (39). We therefore addressed the role of intact p53 in A-1331852 response. We first evaluated p53 mutation status in our panel of ccRCC lines, as described in the Broad Institute’s Cancer Cell Line Encyclopedia (CCLE). This analysis showed that wild-type p53 was present in 3 of the 4 BCL-X_L_-dependent lines (e.g. CAKI-2, A498, and TUHR4TKB), but also in two of the BCL-X_L_ independent cell lines (e.g. OSRC2 and UOK101) (Supplementary Fig. S4A). We then treated ccRCC lines with doxorubicin and measured the protein levels of total p53, phosphorylated p53, and the p53-target gene *CDKN1A*, which encodes the cyclin-dependent kinase inhibitor p21. We found that (with the exception of UOK101 cells) the basal levels of p21, and its induction in response to doxorubicin, was lower in many of the intermediate and insensitive ccRCC lines compared with the sensitive cells (Supplementary Fig. S4B). Lastly, using isogenic ccRCC cells, where we first inactivated endogenous p53 using CRISPR/Cas9, and then restored expression of sgRNA-resistant versions of either wild-type or mutant p53, we found that changes in p53 activity had no discernible impact on A-1331852 response (Supplementary Fig. S4, C – E). Altogether, we concluded that intact p53 response was largely insufficient to predict BCL-X_L_ dependency in ccRCC lines.

We then focused our attention on epithelial-mesenchymal transition (EMT) as a determinant of BCL-X_L_ dependency. One previous study had suggested this possibility, albeit in cancer cells of other lineages (40). Consistent with our RNA-Seq data, using flow cytometry, we noted higher expression levels of the CD44 mesenchymal marker in ccRCC cells that were sensitive to acute BCL-X_L_ inhibition (Fig. 3E).

**FIGURE 4.**
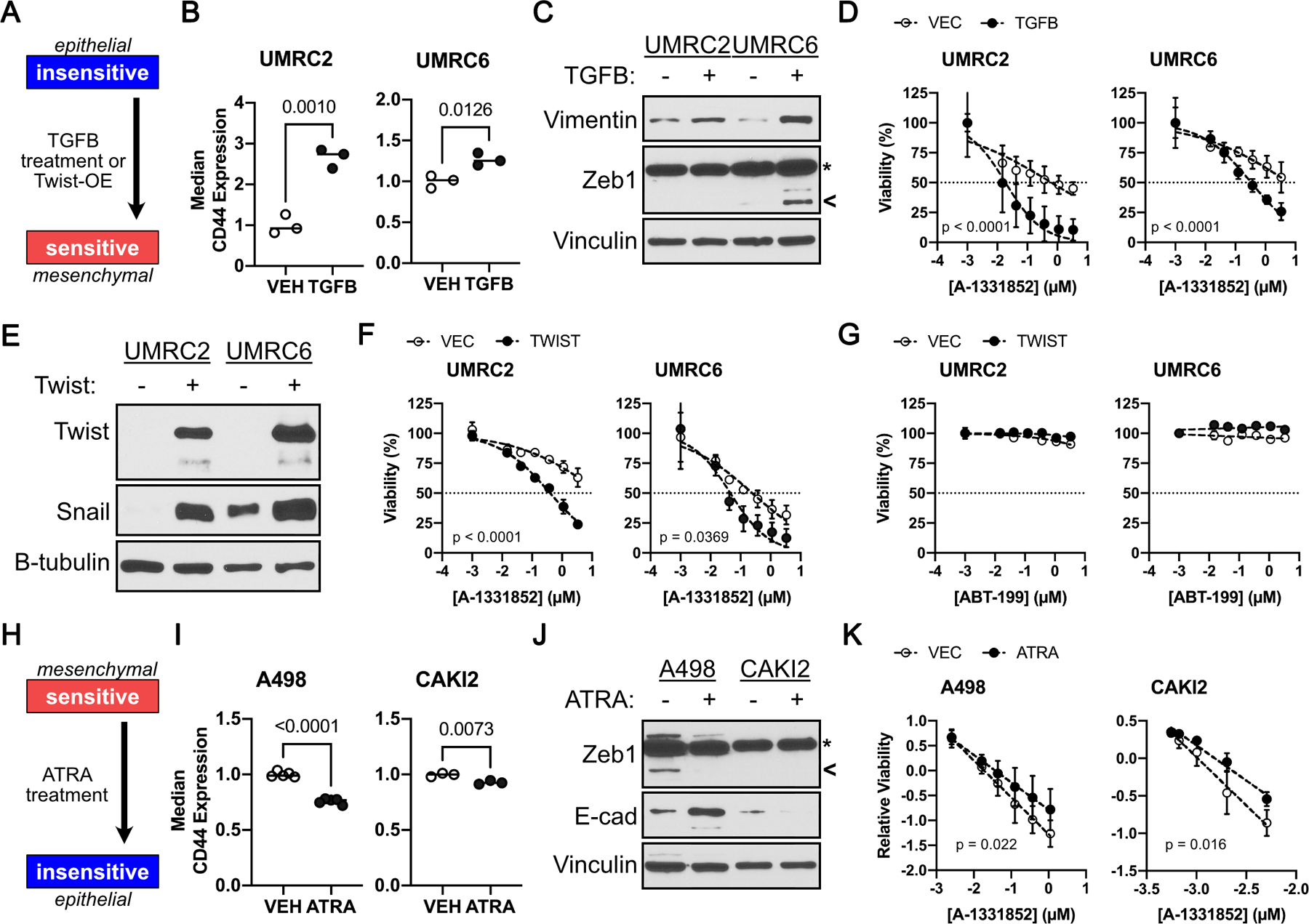
A Mesenchymal Cell State Promotes BCL-X_L_ Dependency. **A,** Schema showing experimental design to address sufficiency of mesenchymal state in promoting Bcl-xL dependency. CD44 levels, as determined by flow cytometry (**B**), and immunoblot analysis of the indicated proteins (**C**), in A-1331852 insensitive ccRCC cells that were treated with 10 ng/ml TGFβ for 3 days. **D,** Cell viability, as measured using CellTiter-Glo, in the indicated cells treated with A-1331852 for 7 days. Immunoblot analysis (**E**) and cell viability, as measured by Cell-TiterGlo, in the indicated insensitive ccRCC cells that were treated with the indicated concentration of A-1331852 (**F**) or ABT-199 (**G**) for 7 days. (**H**) Schema showing experimental design to address necessity of mesenchymal state in promoting BCL-X_L_ dependency. CD44 levels, as determined by flow cytometry (**I**), and immunoblot analysis (**J**), in the indicated ccRCC cells that were treated with 1 µM ATRA for 3 days. **K,** Cell viability, relative to untreated DMSO controls, in the indicated cells treated with A-1331852 for 7 days. Data in (**B**) and (**E**) was normalized to CD44 levels in the untreated (Vehicle) control and was compared using the Student’s t-test (n ≥ 3, bar represents Mean, p-values are indicated). In (**D**), (**F**), (**G**), and (**K**), data were normalized for batch effects and compared using linear regression (n ≥ 3, mean±S.D., line represents best fit, p-values indicated are for difference in slopes i.e., interaction between A-1331852 and ATRA or TGFβ. In (**D**), (**F**), (**G**), and (**K**), all data points were plotted relative to the untreated DMSO controls in the given experimental arm, and all concentrations are presented as log_10_. In (**C**) and (**J**) arrowhead marks the ZEB1 band; whereas, asterisk marks a non-specific band, which doesn’t respond to cell state modifiers.

Next, we addressed the contribution of EMT in driving apoptosis upon A-1331852 treatment. We began by treating the A-1331852-insensitive, UMRC-2 and OSRC-2, cells for 3 days with TGFβ, a potent inducer of mesenchymal transition (Fig. 4A). Unfortunately, we did not find any changes in CD44 expression in OSRC2 cells and thus substituted these cells with the intermediate-sensitivity UMRC-6 cells. In both UMRC-2 and UMRC-6 cells, TGFβ treatment led to induction of mesenchymal features (e.g. CD44, Vimentin, and Zeb1) (Fig. 4B and C) and concurrently increased A-1331852 sensitivity (Fig. 4D). We then lentivirally expressed the EMT master transcription factor, TWIST, in UMRC-2 and UMRC-6 cells. Here again, we observed that inducing mesenchymal features (e.g. TWIST and SNAIL) (Fig. 4E) increased sensitivity to A-1331852 (Fig. 4F). Importantly, these results were BCL-X_L_ specific because mesenchymal transition failed to sensitize ccRCCs to pharmacological BCL-2 inhibition (Fig. 4G).

Finally, we addressed mesenchymal necessity (Fig. 4H). Treating the sensitive lines, A-498 and CAKI-2, with all-trans-Retinoic Acid (ATRA) for three days led to a small, but measurable, decrease in mesenchymal features (e.g. CD44 and Zeb1 decreased; whereas, E-cadherin increased) (Fig. 4I and J). Interestingly, this minimal change was sufficient to modestly reduce the sensitivity of both A-498 and CAKI-2 cells to A-1331852 treatment (Fig. 4K). Altogether, EMT was both necessary and sufficient to confer BCL-X_L_ dependence in ccRCCs.

### Genetic Hallmarks of ccRCC do not Confer BCL-X_L_ Dependency

An examination of the ccRCC cells indicated that recurrent genetic hallmarks of ccRCC, including loss of *VHL*, *PBRM1*, *BAP1*, *SETD2*, etc (2), were insufficient to predict BCL-X_L_ dependence (Supplementary Fig. S4F). Next, because pVHL expression can impact EMT in certain cell lines, (41, 42), we addressed if pVHL status influences BCL-X_L_ dependency. Using isogenic pVHL-proficient or –deficient versions of CAKI-2 and A-498 cells (Supplementary Fig. S4G), we found that, surprisingly, reintroduction of pVHL failed to alter their mesenchymal cell state (Supplementary Fig. S4H) and did not impact their response to pharmacological BCL-X_L_ inhibition (Supplementary Figs. S4I and S4J).

ccRCCs vary in their HIFα expression patterns, either expressing HIF2α alone (H2) or both HIF1α and HIF2α (H1H2)] (43, 44). HIFα status can impact certain biological outcomes, such as response to HIF2α blockade (10). However, we found that A-1331852-sensitive ccRCC cells included both H2 (e.g. A-498) and H1H2 (e.g. CAKI-2) cell lines. Conversely, cell lines that were insensitive to BCL-X_L_ loss also included H2 (e.g. UOK101) and H1H2 (e.g. UMRC-2, RCC4, etc) cells. Together, these results indicated that (1) the impact of pVHL on EMT was likely dependent on biological context; and, (2) neither pVHL nor HIFα status was sufficient to predict BCL-X_L_ dependence.

### The ‘BCL-X_L_ Dependence’ Signature is a Clinically-exploitable Feature

**FIGURE 5.**
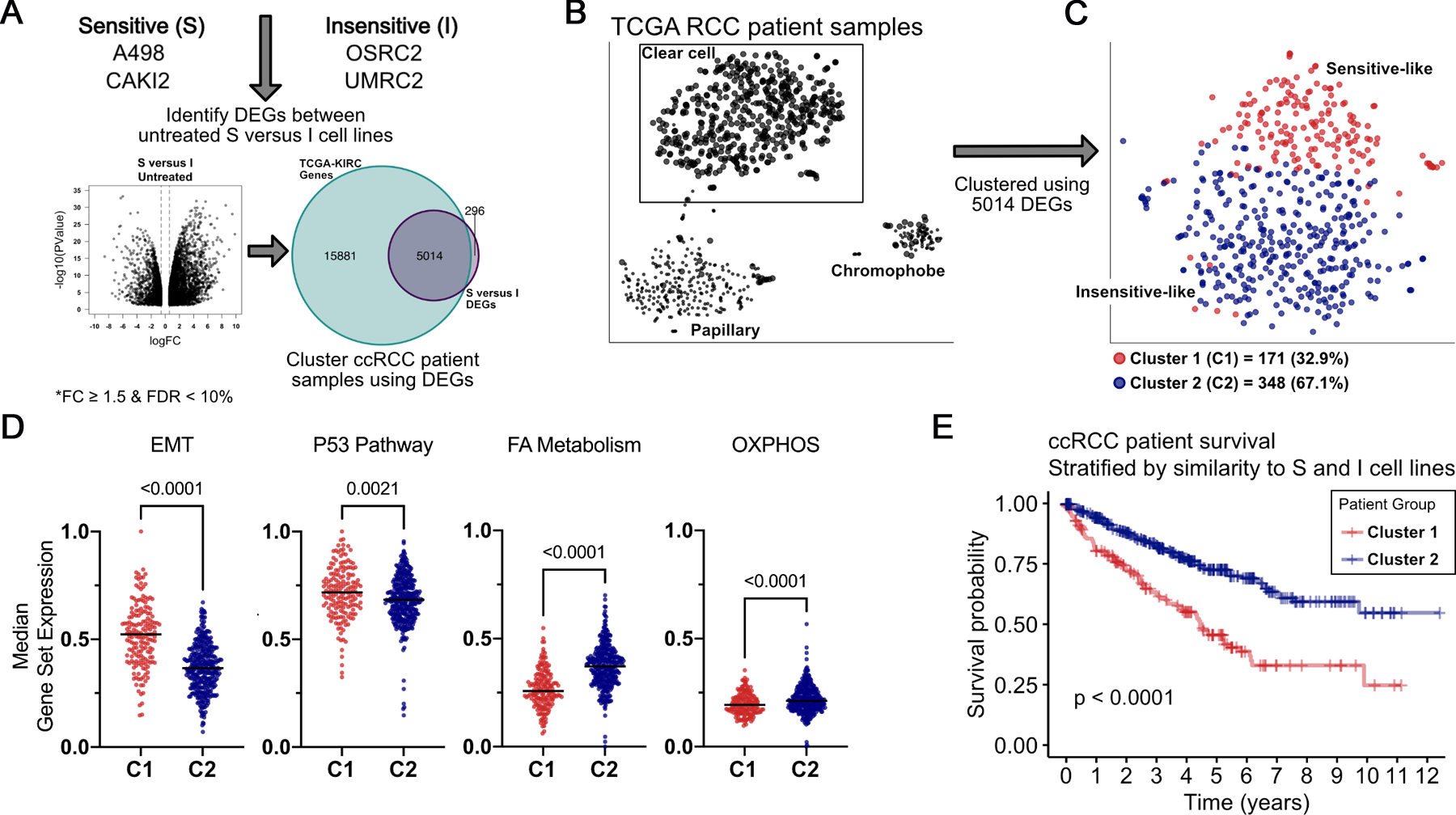
BCL-X_L_ Dependency Signatures are Evident in ccRCC Clinical Specimens. **A,** Schema indicating the computational overlay of differentially-expressed genes, as determined in 4A, onto renal cancer gene expression data mined from TCGA. **B,** Principle-component analysis showing segregation of tumors based on their subtype (left), and then within ccRCC (**C**) by similarity in gene signature to Sensitive (red) versus insensitive (blue) lines (right). Median gene expression levels of the indicated gene sets (**D**) and Kaplan-Meier curves depicting clinical outcomes (**E**) in patients with tumors resembling the BCL-X_L_ inhibitor Sensitive versus Insensitive cell lines. (**A**) and (**D**) were compared using the Student’s *t test*. In (**E**) Sensitive (n = 171) and Insensitive (n = 348) patients were compared by the log-rank test.

Transcriptional differences identified determinants of BCL-X_L_ dependence in ccRCC cells; therefore, we used DEGs (identified in Fig. 3) to probe for the prevalence of these signatures in human RCC tumor gene expression data from The Cancer Genome Atlas (TCGA) (Fig. 5A). Principal component analysis segregated RCC tumors into three clusters, each representing one major RCC disease subtype (Fig. 5B; Supplementary Fig. S5). Overlaying the DEGs predicting BCL-X_L_ dependence onto human ccRCC tumors allowed us to establish that (**a**) the BCL-X_L_ dependency signature (red) was observed in ~30% of human ccRCCs (Fig. 5C); and, (**b**) the EMT signature was among the most differentially expressed gene signatures among these cohorts. Differences in the p53 pathway, albeit statistically significant, showed more subtle differences (Fig. 5D). Finally, as expected from the typically aggressive nature of mesenchymal tumors, the BCL-X_L_ dependency signature was associated with worse clinical outcomes (Fig. 5E). These studies suggested that mRNA gene expression could be exploited as a biomarker to identify patient cohorts that exhibit features of BCL-X_L_ dependence.

### BCL-X_L_ Inhibition Impedes *In Vivo* Tumor Growth

The BCL-X_L_-selective inhibitor, A-1331852, is orally bioavailable in subcutaneous tumor xenografts in NCR^nu/nu^ mice (23), and could be used to interrogate the therapeutic relevance of BCL-X_L_ inhibition in ccRCC. Unfortunately, the BCL-X_L_-dependent CAKI-2 and A-498 cells, have poor engraftment rates as subcutaneous tumors in NCR^nu/nu^ mice. The UMRC-2 cells, however, engraft tumors readily. Interestingly, we found that, as seen with our genetic studies, sustained pharmacological BCL-X_L_ inhibition (>2 weeks) led to nM IC_50_ in UMRC-2 cells (Supplementary Fig. S6), justifying the use of UMRC-2 cells for *in vivo* studies, which typically rely on dosing regimens extending to 3–4 weeks.

**Figure 6.**
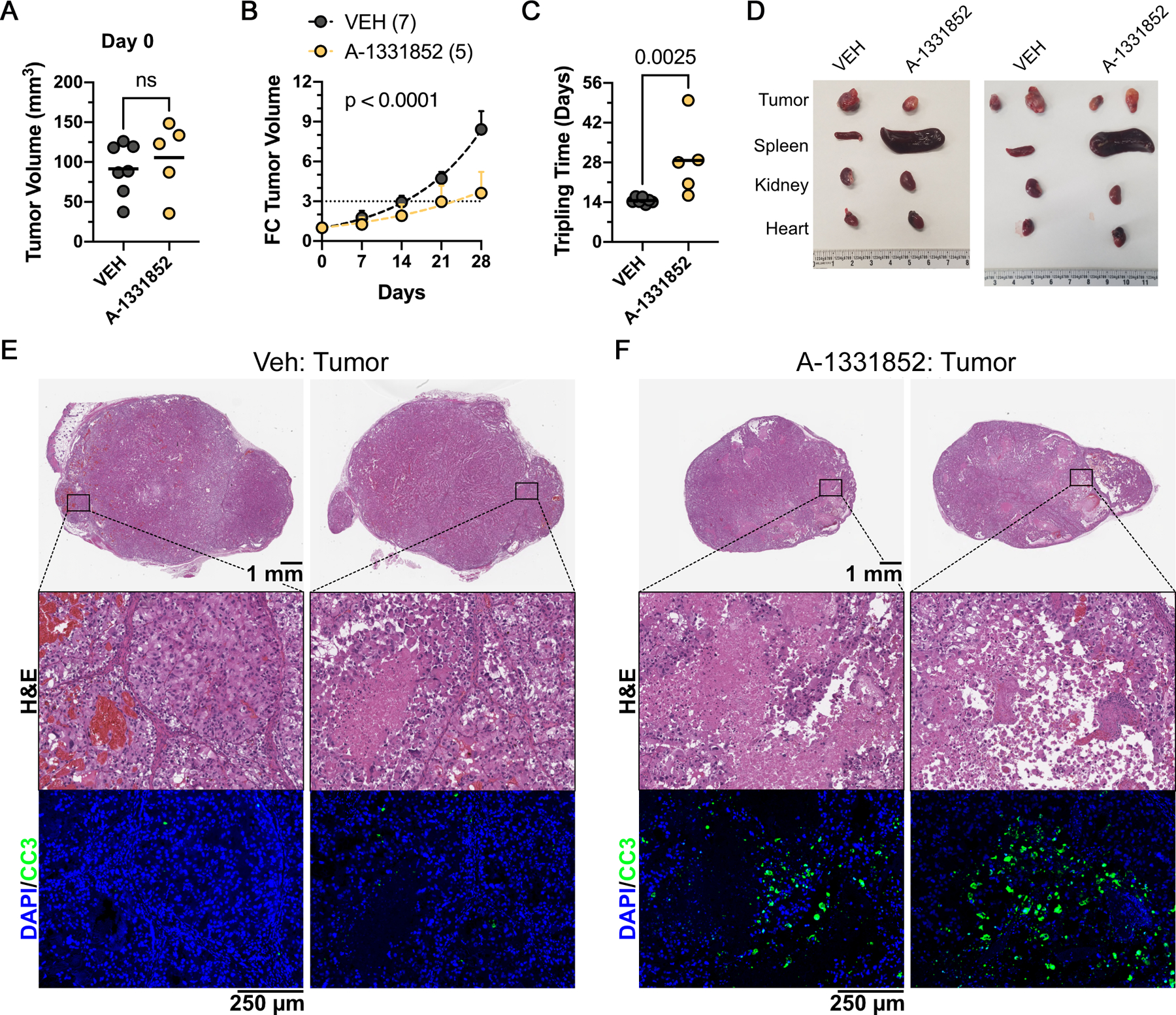
Pharmacological BCL-X_L_ Inhibition Impedes Tumor Growth. Tumor volumes at Day 0, start of dosing (**A**), change in tumor volume at the indicated time points after A-1331852 dosing at 25 mg/kg, twice a day, by oral gavage (**B**), time for tumor volume to triple on the indicated dosing regimens (**C**), and photomicrographs of harvested tumors and the indicated animal organs (**D**), in NCR^nu/nu^ mice that were inoculated subcutaneously with pVHL-deficient UMRC-2 cells. Histological analysis by H&E staining and immunohistochemistry, showing DAPI (blue) and Cleaved Caspase 3 (green) staining (bottom panel), of tumors harvested from animals dosed with Vehicle control (**E**) or A-1331852 (**F**), as indicated.

We engrafted UMRC-2 cells into subcutaneous tumors in 7 week old NCR^nu/nu^ mice (equal number of males and females). Tumor-bearing animals (>100 mm^3^) were randomized to receive either 25 mg/kg A-1331852 or sham-vehicle control, twice a day, orally, for up to 4 weeks. Compared to the indistinguishable tumor volumes at the point of enrollment (Fig. 6A), we noted smaller tumor volumes and slower tripling rates in A-1331852 treated mice (Fig. 6, B – D). Finally, histological analysis of the harvested tumors showed zones of dying cells, marked by elevated Cleaved Caspase 3 staining, consistent with apoptotic cell death, in A-1331852 treated tumors (Fig. 6E and F).

Despite no notable behavioral or body weight changes (Supplementary Fig. S7A), we found profound splenomegaly and a disruption of splenic tissue architecture in A-1331852 treated mice (Fig. 6D; Supplementary Fig. S7, B – D), but no changes in other major organs (e.g. kidney, liver, lung, and heart; Supplementary Fig. S8). Altogether, these findings were promising. Even with the technical necessity to employ the relatively insensitive UMRC-2 cells, we demonstrated the overall feasibility of BCL-X_L_ inhibition as a strategy to block ccRCC tumor growth.

## DISCUSSION

Despite recent therapeutic advances, metastatic kidney cancer is ultimately incurable and new therapies that complement existing treatments are sorely needed. To address this need, instead of ‘synthetic lethal’ dependencies that are associated with a specific oncogenic alteration (e.g. truncal genetic lesion), we interrogated kidney lineage-specific dependencies. Second, within the lineage, we sorted for strongly dependencies even when restricted to a subset of cells. This strategy enabled the identification of ~twenty candidate “hits”, including the anti-apoptotic BCL-X_L_ protein.

We focused on BCL-X_L_ because it already had well characterized pharmacological inhibitors available, which could facilitate faster adaptation into the clinic. However, other genes in our list (e.g. *ITGAV*) also encode druggable proteins and warrant further study. Moreover, some otherwise “undruggable” proteins (e.g. HNF1B and PAX8) could be targeted with drugs that promote their proteolysis, such as deubiquitinase inhibitors and/or proteolysis-targeting chimeras (PROTACs). Notably, PROTACs have also been developed for BCL-X_L_, in an attempt to reduce drug resistance and to reprogram BCL-X_L_ inhibition away from platelets, where it leads to undesirable cytotoxic side effects (45).

We rigorously validated BCL-X_L_ as a dependency in ccRCC. First, we confirmed that ccRCC lines, such as CAKI-2 and TUHR4TKB, which were exceptionally sensitive to *BCL2L1* in the shRNA dataset (13), were also identified as BCL-X_L_ dependent in the Broad Institute’s CRISPR/Cas9 dependency map (21Q3) (14). Next, using three different shRNAs, we confirmed fitness defects in multiple ccRCC cell lines upon loss of BCL-X_L_, but not its closely related sibling BCL-2. Finally, we demonstrated that these effects were “on-target” by rescuing the cytotoxicity associated with two BCL-X_L_ shRNAs.

To develop our genetic studies, we employed rigorous pharmacological studies. We found remarkable overlap in the BCL-X_L_ dependence of cell lines in both of these approaches. We also noted that some cell lines (e.g. UMRC-2) showed cytotoxicity only upon chronic BCL-X_L_ blockade. BCL-2 loss was well tolerated by ccRCCs. Finally, A-1331852’s cytotoxicity could be partially reversed using ‘drug-resistant’ BCL-X_L_ mutants. These findings demonstrate the specificity of BCL-X_L_ dependence in ccRCC.

Standard chemotherapeutic agents, including topoisomerase inhibitors, taxanes, and nucleoside analogues, have failed in many independent trials against ccRCC (33). The ability to promote apoptosis is central to the efficacy of many traditional chemotherapeutic agents. Our drug-drug interaction studies show that BCL-X_L_ function presents a physiological barrier to chemotherapeutic response in kidney cancer.

The importance of anti-apoptotic proteins has been previously interrogated in renal cancer; however, these studies focused primarily on the role of BCL-2 (46–48). Our findings demonstrate that ccRCCs exhibit much greater dependence on BCL-X_L_ than BCL-2. These findings refocus attention on BCL-X_L_ blockers as potential therapeutic agents in kidney cancer.

Our findings indicate that ~30% of human renal tumors, especially those that represent the more aggressive mesenchymal signatures, are likely to be responsive to BCL-X_L_ inhibition. We also demonstrate the utility of the *ex vivo* BH3-profiling assay as a faithful predictor of BCL-X_L_ dependency in renal cells. Importantly, this assay has been optimized for interrogation of apoptotic dependencies in human cancer samples (32, 49), and can be easily adapted for human renal tumors. Together, these assays provide us future opportunities, which were beyond the scope of this initial study, to develop clinically usable biomarkers to identify cohorts of BCL-X_L_ dependent renal tumors.

The clinical use of BCL-X_L_ blockers has been limited due to adverse events (e.g. thrombocytopenia). However, most of these studies were performed using earlier versions of BCL-X_L_ blockers, which also had ‘off-target’ effects on other BCL-2 family proteins. Specific BCL-X_L_ inhibitors, such as A-1331852, may have reduced toxicities *in vivo*. Thrombocytopenia will continue to be a concern given that platelets are highly dependent on BCL-X_L_ for survival and quickly undergo apoptosis when this protein is inhibited (50). However, novel approaches that degrade BCL-X_L_ in nucleated cells, but not platelets, reduce thrombocytopenia in animal models, and are now entering clinical trials (45). These pharmacological advances, combined with assays that predict BCL-X_L_ dependence, and assign therapy more rationally in patients most likely to respond, could enable efficacious clinical use of BCL-X_L_ inhibitors in kidney cancer.

## Supplementary Material

1

2

3

4

5

6

7

8

## TRANSLATIONAL RELEVANCE

clear cell Renal Cell Carcinomas (ccRCCs) represent the majority of adult kidney cancers. Advanced/metastatic ccRCC remains incurable, highlighting the need for new therapies. To address this need, we exploited the availability of genome-wide genetic dependency maps and identified the BCL-X_L_ anti-apoptotic protein as an actionable target in kidney cancer. We then functionally validated (both *in vitro* and *in vivo*), measured drug-drug interactions with standard chemotherapeutics, identified biomarkers, and established the cellular basis of this BCL-X_L_ dependency. Using clinical datasets, we noted that the biomarkers associated with BCL-X_L_ dependency were observed in ~30% of human ccRCCs, which incidentally were also associated with worse outcomes. Therapeutic agents that specifically target BCL-X_L_ are currently being tested against blood-borne malignancies. Our findings justify testing the utility of BCL-X_L_ blockade in ccRCC and provide the predictive biomarkers that would enable stratification of patients into cohorts that are most likely to benefit from this therapeutic intervention.
