# Supplementary material for: A Mesenchymal Tumor Cell State Confers Increased Dependency on the BCL-XL Anti-apoptotic Protein in Kidney Cancer
Source: Clin Cancer Res. Author manuscript; Available in PMC 2023 May 1. (PMC9633392; doi:10.1158/1078-0432.CCR-22-0669)
Supplement: 1 [file NIHMS1821904-supplement-1.pdf]

SUPPLEMENTARY FIGURE LEGENDS

Fig. S1. Pharmacological Characterization of BCL-X_L Blockade in ccRCC cells. (**A** and **B**) Percent cell death, as determined using XTT assays (**A**) and IC_{50} values modeled using standard regression of log(dose) versus response (**B**), in the indicated ccRCC cell lines. In (**A**) data represents mean±S.D. n ≥ 3. (*****) represents IC_{50} were calculated by extrapolation because cytotoxicity was < 50% even at the highest treated concentration. (**C** and **D**) IC_{50} values modeled using standard regression of log(dose) versus response, from at least 3 independent measurements in the indicated ccRCC cell lines, either using the BCL-X_L inhibitor A1331852 (**C**) or the BCL-2 inhibitor ABT-199 (**D**). ND represents Not Determined, because the cytotoxicity was below 50% death, relative to untreated control. (**E**) Cell viability, as measured by CellTiter-Glo in the indicated cell lines upon treatment with A-1331852 or ABT-199 for 3 days. Concentrations are presented as log_{10}. (**F**) Structural model of BCL-XL in complex with the (A-1331852-precursor) tetrahydroisoquinoline-pyridine backbone (PDB: 6VWC), generated using ChimeraX. (**G**) Immunoblot analysis of UMRC-2 cells that were lentivirally transduced to express sh499-resistant versions of either wild-type BCL-X_L, the indicated BCL-X_L mutants, or empty vector (VEC). (**H**) Crystal violet staining of the indicated cells that were first transduced as in (**G**), and then were lentivirally transduced to express either BCL-X_L targeting shRNA (sh499) or a non-targeting control (shCON), and grown in the presence of puromycin for 7 days. (**I**) IC_{50} values measured from three independent replicates of UMRC-6 cells expressing either wild-type BCL-X_L, the indicated BCL-X_L mutants, or empty vector (VEC), as indicated, and then cultured in the presence of A-1331852 for 3 days. (**J**) Crystal violet staining of UMRC-6 cells, as described in (**I**), that were cultured in the

presence of 2 μM A-1331852 for 10 days.

Fig. S2. BCL-X$_L$ Inhibition Chemosensitizes ccRCCs to Chemotherapeutics. (**A, C, E, G, I,** and **K**) Mean synergism score calculated across four independent models (ZIP, HSA, Loewe, Bliss), using the SynergyFinder package, for 5-fluorouracil (5-FU) (**A** and **C**), docetaxel (**E** and **G**), and doxorubicin (**I** and **K**), used at the indicated doses in combination with A-1331852. **(B, D, F, H, J** and **L)** Percent cell viability, as measured using XTT, and calculated dose-response curves in cells cultured in the presence of the indicated concentrations of the two molecules for 72 hours. Results for UMRC-2 (**A**, **B**, **E**, **F**, **I** and **J**) and OSRC2 (**C**, **D**, **G**, **H**, **K**, and **L**) were calculated from ≥3 biological replicates. Error bars represent mean±SD.

Fig. S3. Transcriptional Determinants of A-1331852 Response. (**A**) Pearson correlation coefficient of BCL-X$_L$ dependency, as indicated by the DEMETER2 score, and *BCL2L1* mRNA expression, as measured by RNA-Seq. Cells, which were used in the Achilles dependency analysis (described in ***Fig. 1***) were annotated by lineage. (**B** to **E**) Immunoblot analysis (**B**), densitometric quantification using ImageJ (**C** and **D**), and statistical analysis of the ratio of BCL-X$_L$:BCL-2 abundance (**E**), in the indicated ccRCC cells, which were annotated as “Sensitive”, “Intermediate”, or “Insensitive” based on their response to acute BCL-X$_L$ inhibition (described in Fig. 2 and fig. S2). In (**E**), ns = not significant. (**F**) Immunoblot analysis of the indicated ccRCC cells that were treated with either 1 μM ATRA or 10 ng/ml TGFβ for 3 days, and normal mouse kidney tissue (Kidney). MCF7 cells, which are known to express the BCL-X$_S$ isoform are used as a positive

control. (**G**) mRNA expression levels, as measured using the RNA-Seq data described in Fig. 3, for the indicated BCL-2 related genes. Black arrows highlight genes whose mRNA levels are elevated in the sensitive (A498 and CAKI-2) cell lines compared to the insensitive (UMRC-2 and OSRC2) cells. (**H** to **J**) Volcano plots showing significant greater transcriptional response in the sensitive (CAKI-2 and A498) cells, compared to the A-1331852 insensitive (UMRC-2 and OSRC2) cell lines (**H**), gene-set enrichment analysis of the A-1331852 responsive gene-sets in the sensitive lines (**I**), and heatmap showing the most significantly altered genes in the sensitive cells (**J**), upon treatment with A-1331852 for 6-16 hours.

Fig. S4. Contribution of the ccRCC's Genetic Hallmarks in A-1331852 Response. (**A**) Annotated genotypes of the indicated cell lines, as described in the Broad Institute's Cancer Cell Line Encyclopedia. (**B**) Immunoblot analysis in the indicated ccRCC cells, which were annotated as "Sensitive", "Intermediate", or "Insensitive" based on their response to acute BCL-X_L inhibition (described in Fig. 2 and fig. S2), after treatment with Doxorubicin for 6 hours. (**C** to **E**) Immunoblot analysis (**C**) and cell viability, as measured in 786O cells (**D**) and OSRC2 cells (**E**) that were edited using CRISPR/Cas9 to knockout endogenous p53 and then lentivirally transduced with sgRNA-resistant versions of either wild-type p53, R248W mutant p53, or empty vector control (EV), and then treated with the indicated doses (presented as log_{10}) of A-1331852 for 3 days. (**F**) Mutation status of the indicated genes, as described in the Broad Institute's Cancer Cell Line Encyclopedia. (*) Historically, 786O was described as a wild-type p53 cell line; however, recently some laboratories have described a clone with mutant p53. The Kaelin laboratory archives,

which was the source of the 786O cells in this study, maintains the original p53-proficient clone. (**G** to **J**) Immunoblots (**G**), CD44 expression, as measured by flow cytometry (**H**), and percent cell death, relative to DMSO-treated control cells, in cell lines that were treated with ABT-263 (**I**) or A-1331852 (**J**), as indicated, for 3 days. Cell proliferation was determined using XTT in the indicated ccRCC lines that were lentivirally transduced to express pVHL (VHL) or empty vector (Vector). In (**I**) and (**J**), data represents mean±S.D, n = 3.

Fig. S5. Principal Component Analysis Predicts a BCL-X_L Dependent Signature in Human ccRCC Tumors. (**A** to **C**) Eigenvalues (**A**), K-means clustering (**B**), and Principal component analysis (**C**) of the entire kidney cancer gene expression dataset mined from TCGA, showing segregation into 3 principal clusters, driven by disease subtype. (**D** to **F**) Eigenvalues (**D**), K-means clustering (**E**), and Principal component analysis (**F**) of gene expression data from clear cell Renal Cell Carcinomas (KIRC in TCGA) overlaid with the differential gene signature (described in Fig. 4) to identify 3 principal clusters within ccRCC. As shown in (**F**), two of these clusters represent ccRCCs that resemble cellular signatures of Bcl-xL inhibitor sensitive (red) and insensitive (blue) cells. The third cluster represents (likely misannotated) KIRCs that resemble chromophobe tumors in gene expression patterns (dark grey in **F**).

Fig. S6. Kinetics of Cytotoxicity in Response to BCL-X$_L$ Inhibition in ccRCC cells. (**A** and **B**) Crystal violet staining (**A**) and Cell viability, as determined by cell counts (**B**), of A-498 following culture in the presence of the indicated concentration of A-1331852 for the indicated times. (**C** and **D**) Crystal violet staining (**C**) and Cell viability, as determined by cell counts (**D**), of UMRC-2 cells following culture in the presence of the indicated concentration of A-1331852 for the indicated times. In (**B**) and (**D**), cells were counted every using the automated ViCell counter (Beckman) and replated in the presence of fresh drug every 3 days.

Fig. S7. Characterization of splenomegaly in A-1331852-treated animals. (**A**) Mean body weight of enrolled mice over the duration of A-1331852 treatment (25 mg/kg, twice a day, Oral gavage). (**B**) Photographs of representative tumor-bearing mice following 4-weeks of twice daily administration of 25 mg/kg A-1331852 or sham-vehicle control. Black arrow indicates the enlarged spleen in the A-1331852 treated mouse. (**C** and **D**) H&E stains of spleens recovered from either sham-vehicle control (**C**) or A-1331852 (**D**) treated mice, as indicated.

Fig. S8. Histological characterization of the impact of A-1331852 treatment. Histological analysis of H&E stained sections of kidney (**A** and **B**), liver (**C** and **D**), lung (**E** and **F**), and heart (**G** and **H**) that were recovered from mice following 4-weeks of twice daily administration of 25 mg/kg A-1331852 or sham-vehicle control, as indicated.

FIGURE S1

A

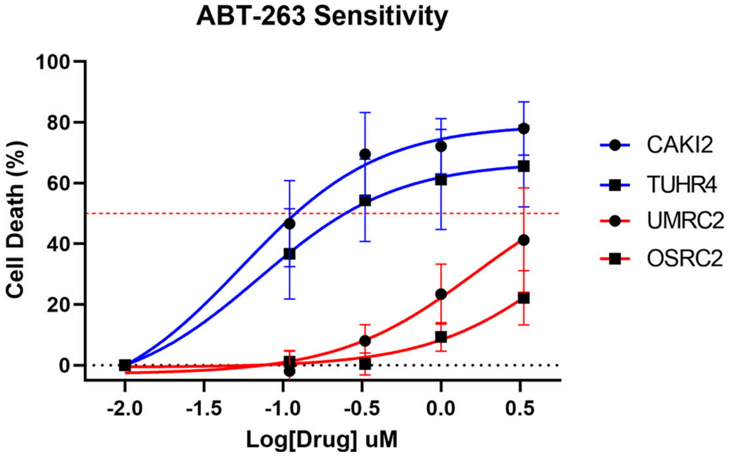

B

Cell Line	IC_{50} (μM)
CAKI2	0.05450
TUHR4	0.07065*
UMRC2	1.629*
OSRC2	6.457

C

Cell Line	A-1331852 IC50 [μM]
CAKI2	0.00069
A498	0.0021
TUHR4	0.017
SLR23	0.012
786O	ND
UMRC6	ND
UMRC2	ND
UOK101	ND
OSRC2	ND
RCC4	ND

D

Cell Line	ABT-199 IC50 [μM]
SUDHL-6	0.063
CAKI2	ND
A498	ND
TUHR4	ND
SLR23	ND
786O	ND
UMRC6	ND
UMRC2	ND
UOK101	ND
OSRC2	ND
RCC4	ND

E

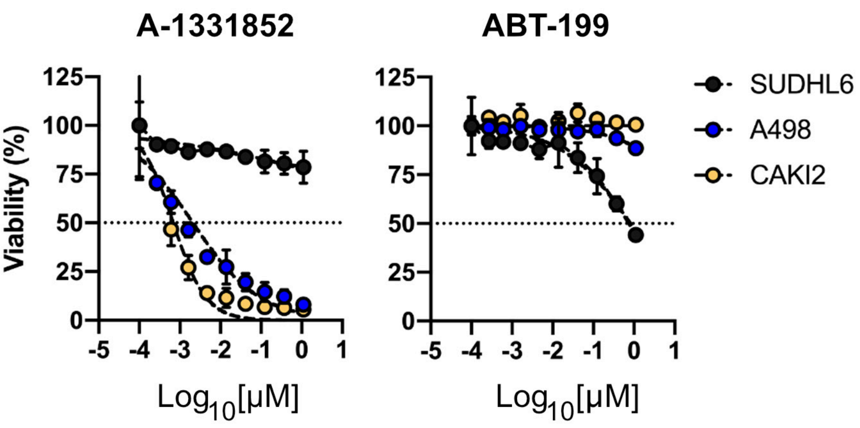

F

A-1331852 in the BCL-X_L binding pocket

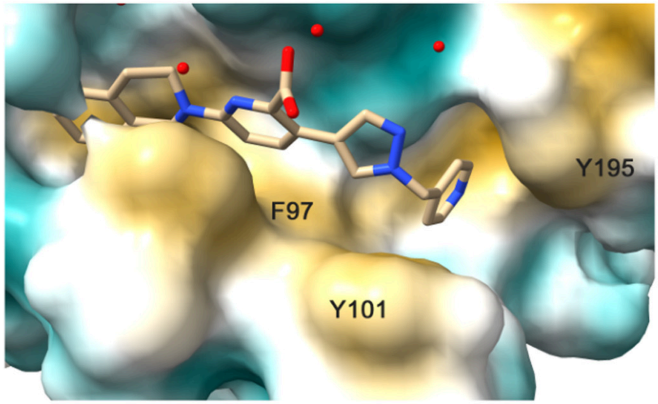

G

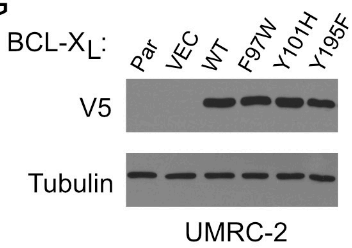

H

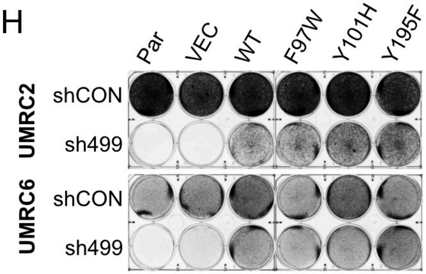

I

BCL-X_L	IC_{50} (μM)
VEC	1.95
WT	1.52
F97W	9.01
Y101H	0.32
Y195F	3.84

J

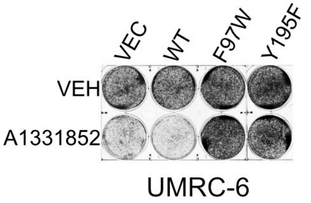

FIGURE S2

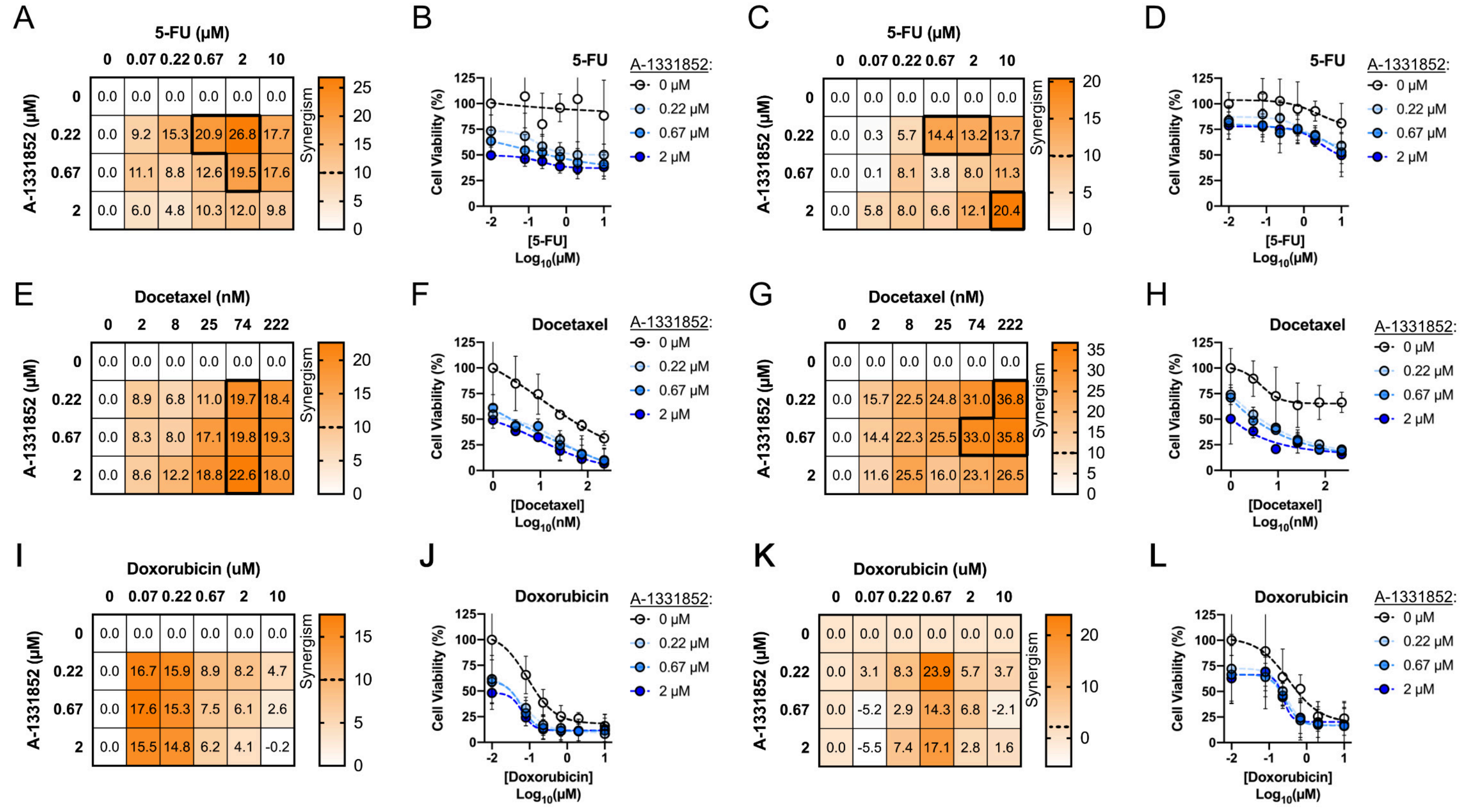

A
5-FU (µM)
0 0.07 0.22 0.67 2 10
A-1331852 (µM)
0: 0.0 0.0 0.0 0.0 0.0 0.0
0.22: 0.0 9.2 15.3 20.9 26.8 17.7
0.67: 0.0 11.1 8.8 12.6 19.5 17.6
2: 0.0 6.0 4.8 10.3 12.0 9.8
Synergism
0 5 10 15 20 25
B
5-FU
Cell Viability (%)
[5-FU] Log10(µM)
A-1331852:
0 µM
0.22 µM
0.67 µM
2 µM
C
5-FU (µM)
0 0.07 0.22 0.67 2 10
A-1331852 (µM)
0: 0.0 0.0 0.0 0.0 0.0 0.0
0.22: 0.0 0.3 5.7 14.4 13.2 13.7
0.67: 0.0 0.1 8.1 3.8 8.0 11.3
2: 0.0 5.8 8.0 6.6 12.1 20.4
Synergism
0 5 10 15 20
D
5-FU
Cell Viability (%)
[5-FU] Log10(µM)
A-1331852:
0 µM
0.22 µM
0.67 µM
2 µM
E
Docetaxel (nM)
0 2 8 25 74 222
A-1331852 (µM)
0: 0.0 0.0 0.0 0.0 0.0 0.0
0.22: 0.0 8.9 6.8 11.0 19.7 18.4
0.67: 0.0 8.3 8.0 17.1 19.8 19.3
2: 0.0 8.6 12.2 18.8 22.6 18.0
Synergism
0 5 10 15 20
F
Docetaxel
Cell Viability (%)
[Docetaxel] Log10(nM)
A-1331852:
0 µM
0.22 µM
0.67 µM
2 µM
G
Docetaxel (nM)
0 2 8 25 74 222
A-1331852 (µM)
0: 0.0 0.0 0.0 0.0 0.0 0.0
0.22: 0.0 15.7 22.5 24.8 31.0 36.8
0.67: 0.0 14.4 22.3 25.5 33.0 35.8
2: 0.0 11.6 25.5 16.0 23.1 26.5
Synergism
0 5 10 15 20 25 30 35
H
Docetaxel
Cell Viability (%)
[Docetaxel] Log10(nM)
A-1331852:
0 µM
0.22 µM
0.67 µM
2 µM
I
Doxorubicin (uM)
0 0.07 0.22 0.67 2 10
A-1331852 (µM)
0: 0.0 0.0 0.0 0.0 0.0 0.0
0.22: 0.0 16.7 15.9 8.9 8.2 4.7
0.67: 0.0 17.6 15.3 7.5 6.1 2.6
2: 0.0 15.5 14.8 6.2 4.1 -0.2
Synergism
0 5 10 15
J
Doxorubicin
Cell Viability (%)
[Doxorubicin] Log10(µM)
A-1331852:
0 µM
0.22 µM
0.67 µM
2 µM
K
Doxorubicin (uM)
0 0.07 0.22 0.67 2 10
A-1331852 (µM)
0: 0.0 0.0 0.0 0.0 0.0 0.0
0.22: 0.0 3.1 8.3 23.9 5.7 3.7
0.67: 0.0 -5.2 2.9 14.3 6.8 -2.1
2: 0.0 -5.5 7.4 17.1 2.8 1.6
Synergism
0 10 20
L
Doxorubicin
Cell Viability (%)
[Doxorubicin] Log10(µM)
A-1331852:
0 µM
0.22 µM
0.67 µM
2 µM

FIGURE S3

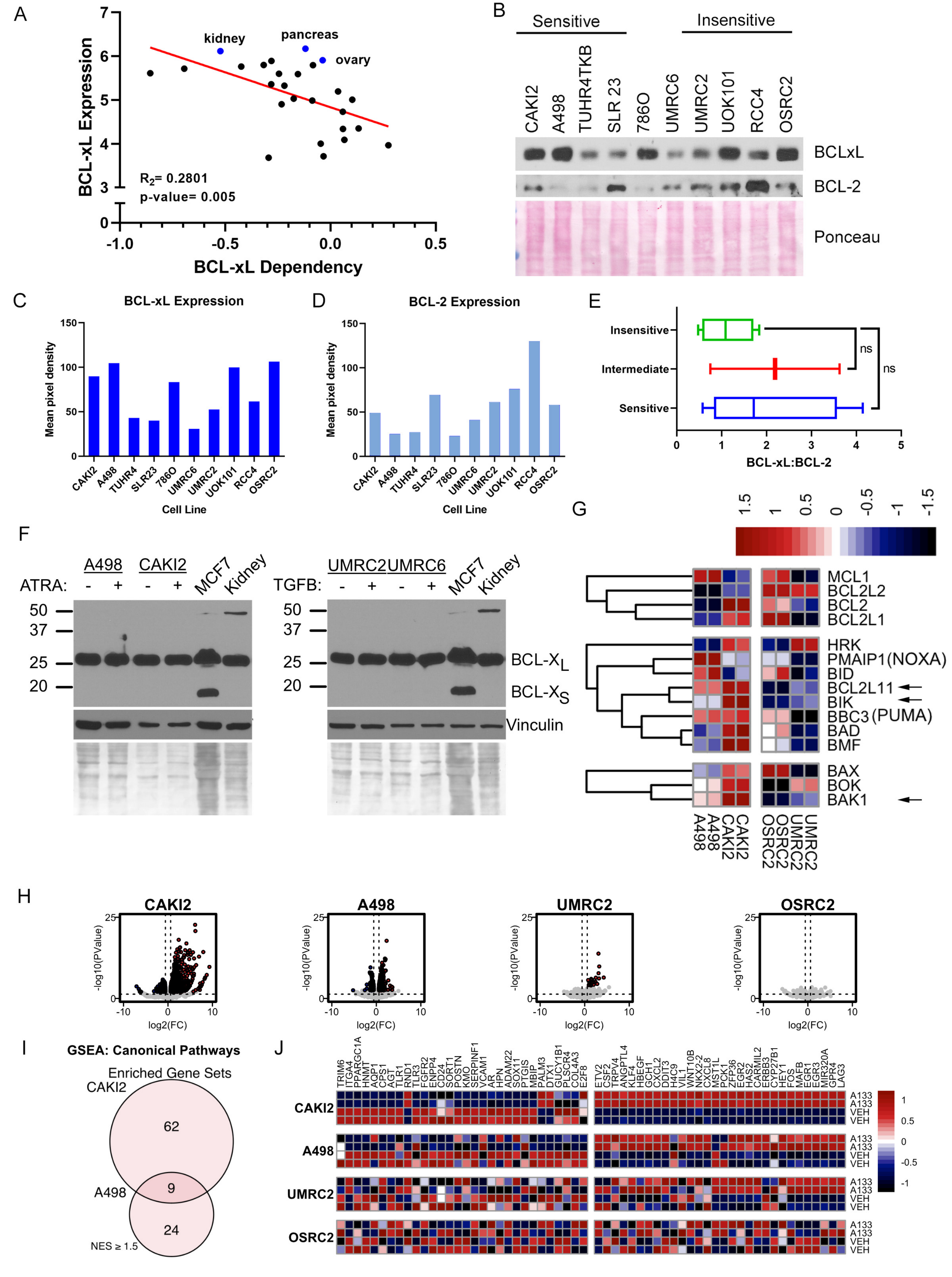
A
BCL-xL Expression
BCL-xL Dependency
kidney
pancreas
ovary
R2= 0.2801
p-value= 0.005
B
Sensitive
Insensitive
CAKI2
A498
TUHR4TKB
SLR 23
786O
UMRC6
UMRC2
UOK101
RCC4
OSRC2
BCLxL
BCL-2
Ponceau
C
BCL-xL Expression
D
BCL-2 Expression
Mean pixel density
Cell Line
E
Insensitive
Intermediate
Sensitive
ns
ns
BCL-xL:BCL-2
F
A498
CAKI2
MCF7
Kidney
ATRA:
UMRC2
UMRC6
TGFB:
BCL-XL
BCL-XS
Vinculin
G
MCL1
BCL2L2
BCL2
BCL2L1
HRK
PMAIP1(NOXA)
BID
BCL2L11
BIK
BBC3(PUMA)
BAD
BMF
BAX
BOK
BAK1
H
CAKI2
A498
UMRC2
OSRC2
-log10(PValue)
log2(FC)
I
GSEA: Canonical Pathways
Enriched Gene Sets
CAKI2
62
9
A498
24
NES ≥ 1.5
J
CAKI2
A498
UMRC2
OSRC2
A133
VEH

FIGURE S4

A

	BCL-xL dep.	TP53	VHL
CAKI2	HIGH	Wild-type	R177*
A498	HIGH	Wild-type	VD142fs
TUHR4TKB	HIGH	Wild-type	Y98fs
SLR23	HIGH	R158H	W88*
786O	INTERMED.	R248W	G104fs
		R278A	
		Splice Site	
UMRC6	INTERMED.		
UOK101	LOW	Wild Type	T157fs
RCC4RGB	LOW		
UMRC2	LOW		
OSRC2	LOW	Wild Type	R58fs

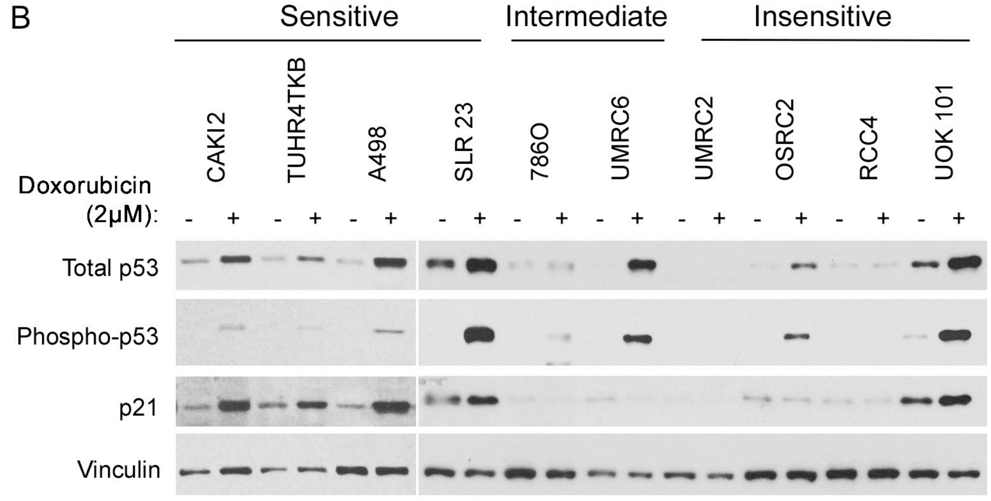

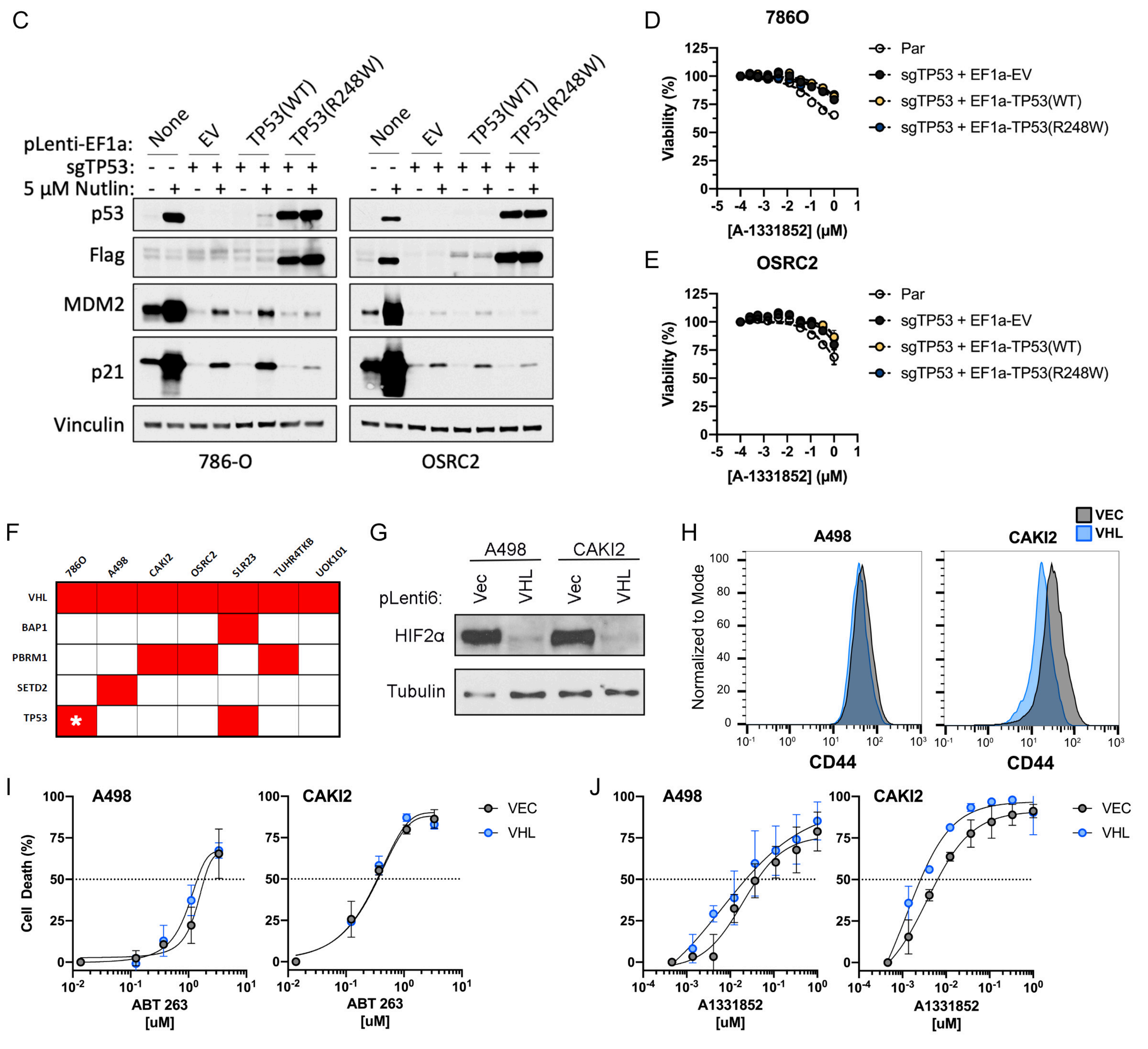

FIGURE S5

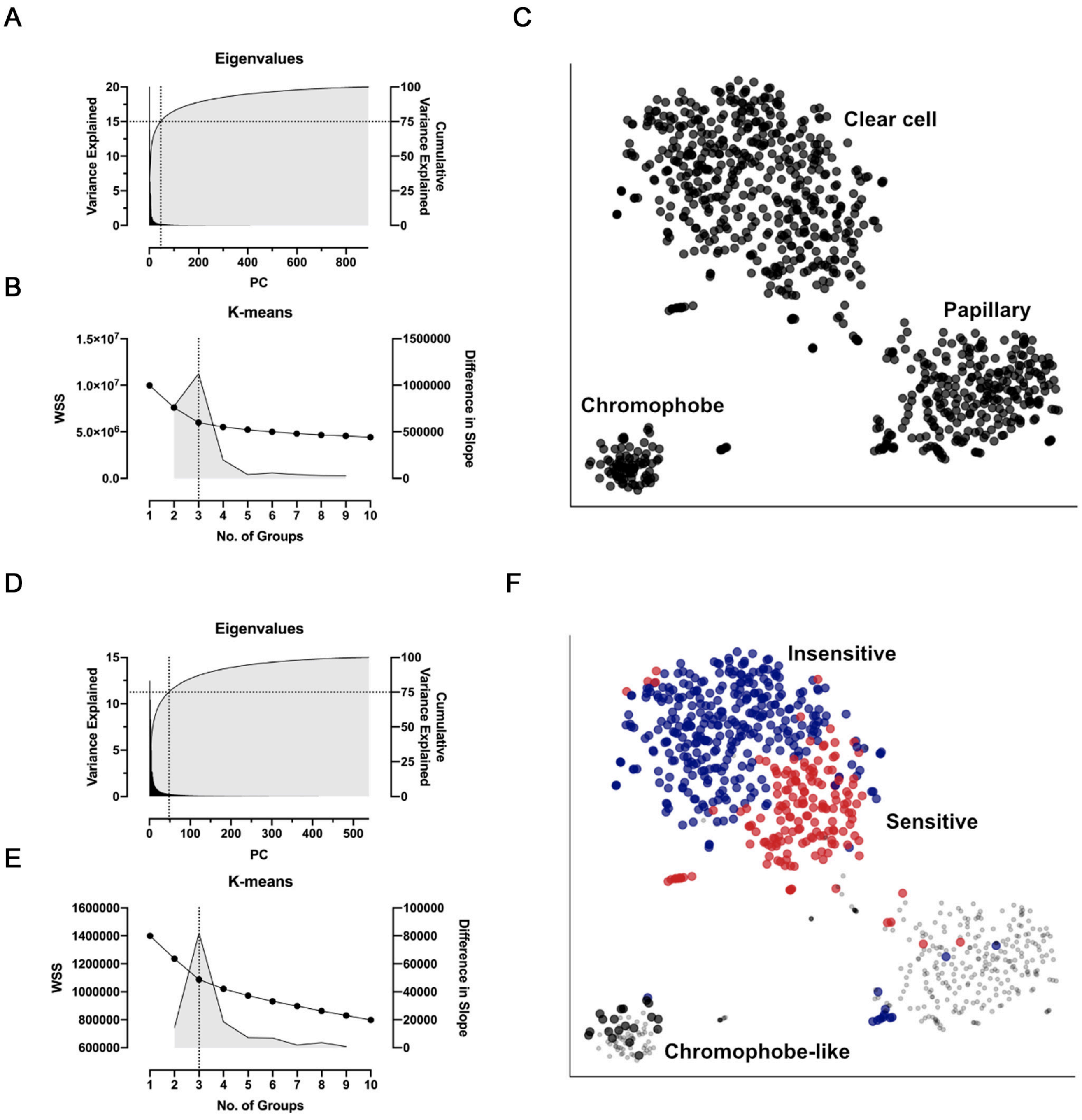
A
Eigenvalues
Variance Explained
Cumulative Variance Explained
PC
0
200
400
600
800
0
5
10
15
20
25
50
75
100
B
K-means
WSS
Difference in Slope
No. of Groups
1.5×10^7
1.0×10^7
5.0×10^6
0.0
1500000
1000000
500000
1
2
3
4
5
6
7
8
9
10
C
Clear cell
Papillary
Chromophobe
D
Eigenvalues
Variance Explained
Cumulative Variance Explained
PC
0
100
200
300
400
500
E
K-means
WSS
Difference in Slope
No. of Groups
1600000
1400000
1200000
1000000
800000
600000
100000
80000
60000
40000
20000
F
Insensitive
Sensitive
Chromophobe-like

FIGURE S6

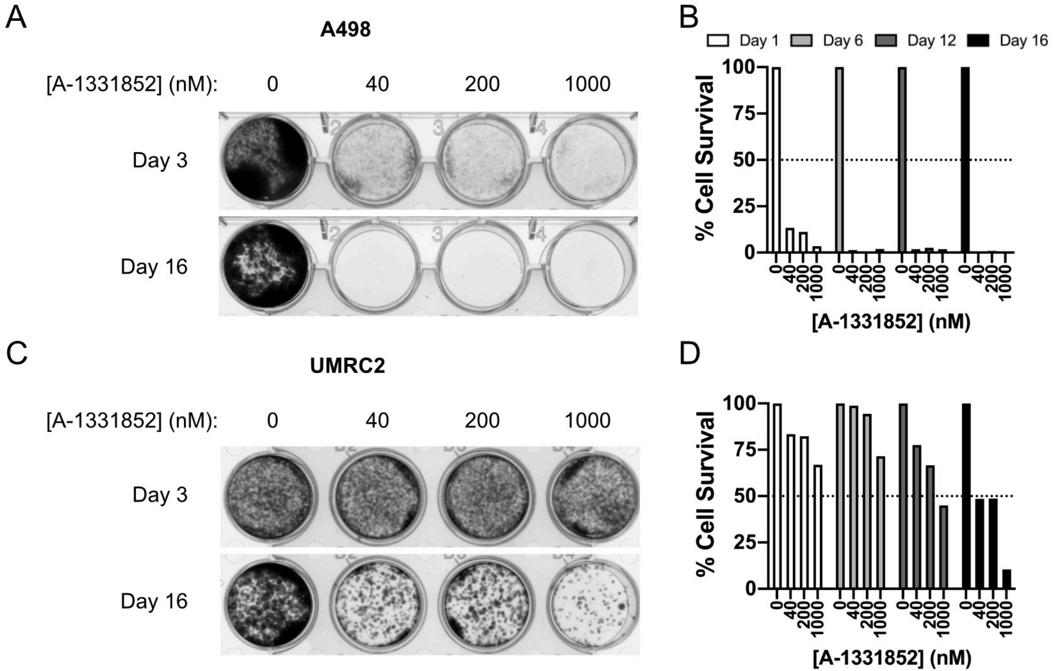
A
A498
[A-1331852] (nM): 0 40 200 1000
Day 3
Day 16
B
Day 1
Day 6
Day 12
Day 16
% Cell Survival
100
75
50
25
0
0 40 200 1000
[A-1331852] (nM)
C
UMRC2
[A-1331852] (nM): 0 40 200 1000
Day 3
Day 16
D
% Cell Survival
100
75
50
25
0
0 40 200 1000
[A-1331852] (nM)

FIGURE S7

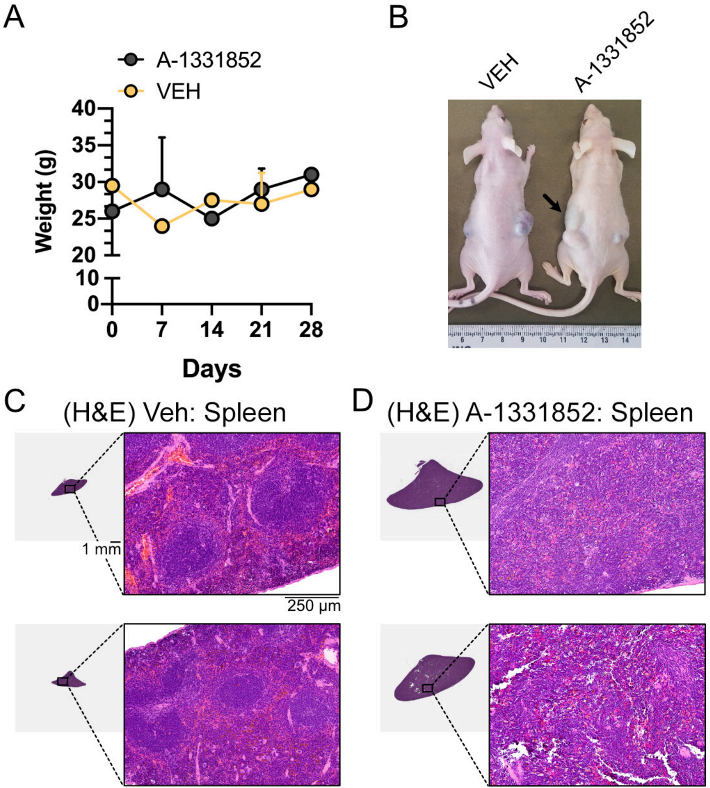
A
A-1331852
VEH
Weight (g)
40
35
30
25
20
10
0
0
7
14
21
28
Days
B
VEH
A-1331852
C
(H&E) Veh: Spleen
1 mm
250 µm
D
(H&E) A-1331852: Spleen

FIGURE S8

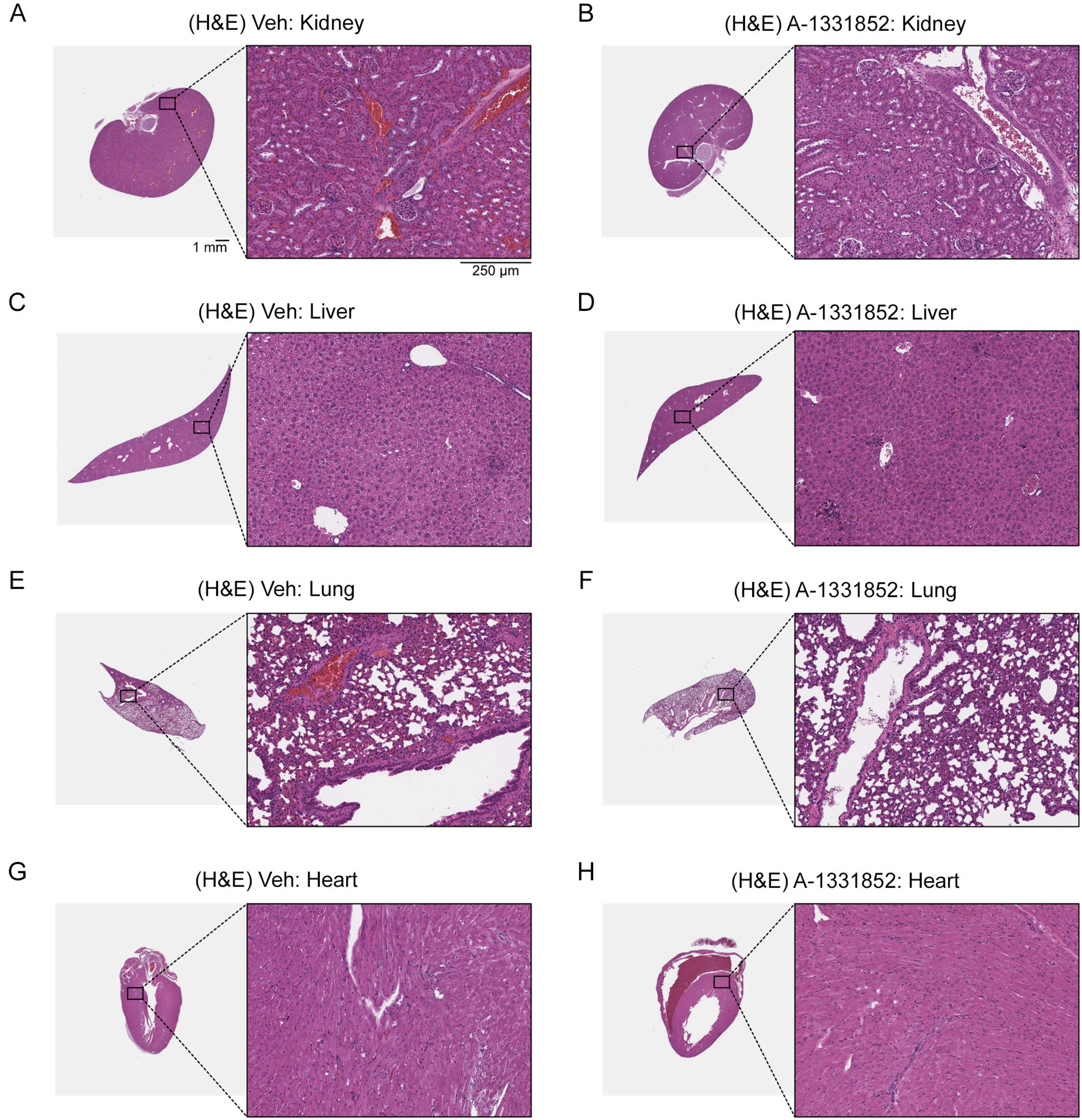
A
(H&E) Veh: Kidney
1 mm
250 µm
B
(H&E) A-1331852: Kidney
C
(H&E) Veh: Liver
D
(H&E) A-1331852: Liver
E
(H&E) Veh: Lung
F
(H&E) A-1331852: Lung
G
(H&E) Veh: Heart
H
(H&E) A-1331852: Heart
